# Intragenic sequences in the trophectoderm harbour the greatest proportion of methylation errors in day 17 bovine conceptuses generated using assisted reproductive technologies

**DOI:** 10.1186/s12864-018-4818-3

**Published:** 2018-06-05

**Authors:** Alan M. O’Doherty, Paul McGettigan, Rachelle E. Irwin, David A. Magee, Dominic Gagne, Eric Fournier, Abdullah Al-Naib, Marc-André Sirard, Colum P. Walsh, Claude Robert, Trudee Fair

**Affiliations:** 1 School of Agriculture and Food Science and Lyons Research Farm, University College Dublin, Belfield, Dublin 4 Ireland; 2 Biomedical Sciences Research Institute, University of Ulster, Coleraine, UK; 3 Centre de Recherche en Biologie de la Reproduction (CRBR), Département des Sciences Animales, Université Laval, Québec, Qc Canada; 4 Department of Animal and Poultry Science, School of Agriculture, Virginia Polytechnic Institute and State University, Blacksberg, VA USA

**Keywords:** Assisted reproduction technologies (ART), Epigenetics, DNA methylation, Embryo, Gene body, Bovine, Genomic imprinting, Reproduction, Development

## Abstract

**Background:**

Assisted reproductive technologies (ART) are widely used to treat fertility issues in humans and for the production of embryos in mammalian livestock. The use of these techniques, however, is not without consequence as they are often associated with inauspicious pre- and postnatal outcomes including premature birth, intrauterine growth restriction and increased incidence of epigenetic disorders in human and large offspring syndrome in cattle. Here, global DNA methylation profiles in the trophectoderm and embryonic discs of in vitro produced (IVP), superovulation-derived (SOV) and unstimulated, synchronised control day 17 bovine conceptuses (herein referred to as AI) were interrogated using the EmbryoGENE DNA Methylation Array (EDMA). Pyrosequencing was used to validate four loci identified as differentially methylated on the array and to assess the differentially methylated regions (DMRs) of six imprinted genes in these conceptuses. The impact of embryo-production induced DNA methylation aberrations was determined using Ingenuity Pathway Analysis, shedding light on the potential functional consequences of these differences.

**Results:**

Of the total number of differentially methylated loci identified (3140) 77.3 and 22.7% were attributable to SOV and IVP, respectively. Differential methylation was most prominent at intragenic sequences within the trophectoderm of IVP and SOV-derived conceptuses, almost a third (30.8%) of the differentially methylated loci mapped to intragenic regions. Very few differentially methylated loci were detected in embryonic discs (ED); 0.16 and 4.9% of the differentially methylated loci were located in the ED of SOV-derived and IVP conceptuses, respectively. The overall effects of SOV and IVP on the direction of methylation changes were associated with increased methylation; 70.6% of the differentially methylated loci in SOV-derived conceptuses and 57.9% of the loci in IVP-derived conceptuses were more methylated compared to AI-conceptuses. Ontology analysis of probes associated with intragenic sequences suggests enrichment for terms associated with cancer, cell morphology and growth.

**Conclusion:**

By examining (1) the effects of superovulation and (2) the effects of an in vitro system (oocyte maturation, fertilisation and embryo culture) we have identified that the assisted reproduction process of superovulation alone has the largest impact on the DNA methylome of subsequent embryos.

**Electronic supplementary material:**

The online version of this article (10.1186/s12864-018-4818-3) contains supplementary material, which is available to authorized users.

## Background

In mammalian livestock species, embryo transfer and other emerging technologies offer significant opportunities for improvements in reproductive efficiency and genetic selection [1]. Assisted Reproductive Technology (ART) treatments involve the isolation and manipulation of gametes and embryos, such as in vitro maturation (IVM), in vitro fertilization (IVF), intracytoplasmic sperm injection (ICSI), in vitro embryo culture (IVC) and hormonal stimulation (SOV). The long- and short-term implications associated with these technologies are not fully determined; however several studies suggest that they are not without complication [2–6]. Evidence that ARTs are not completely benign exists from analyses of bovine ART-derived embryos, which exhibit differences at morphological, physiological, transcriptional, chromosomal and metabolic levels compared to their in vivo-derived counterparts [7].

Epigenetic mechanisms such as chromatin remodelling, histone modification and DNA methylation are fundamental to successful gametogenesis and are required for normal embryonic progression [2, 8]. Of these, DNA methylation remains the most extensively studied; with previous work demonstrating that the appropriate establishment of DNA methylation patterns in gametes and early embryos is essential for normal development [9]. Genomic imprinting is a process that involves appropriate DNA methylation of differentially methylated regions (DMRs) of the maternal and paternally-derived genomes to facilitate parent-of-origin expression of a cohort of genes, many of which are involved with embryonic growth [10, 11]. Many reports detailing the impact of ARTs on genomic imprinting, specifically DNA methylation at imprinted gene DMRs, suggest ART induces aberrant methylation [12–18], while others indicate that the DMRs remain unaffected [19–22]. Investigations of the epigenetic impact of ovarian stimulation in mouse models indicate that imprint establishment and global methylation status in oocytes is not affected, but that maintenance of imprints post-fertilization is affected [22]. For example, DNA methylation analysis at chromosome 7 in single mouse in vitro cultured blastocysts has shown widespread aberrancies, when compared to in vivo samples [23]. Furthermore, analysis of blastocysts [24], mid-gestation placentas [25] and full term liver and brain tissue [26], derived from superovulated females, indicated altered DNA methylation and/or gene expression at candidate imprinted DMRs. Most recently, findings from an investigation using a mouse model suggest that individual ART procedures cumulatively increase placental morphological abnormalities and epigenetic perturbations [27]. A recent investigation by Saenz-de-Juano et al. demonstrated that embryos developed using an in vitro follicular culture (IFC) method inflicted no additional epigenetic alterations at a small number of imprinted genes (*Snrpn*, *H19* and *Mest*) compared with conventional ovulation induction, suggesting that IFC is a suitable, patient-friendly alternative to ovarian stimulation [28].

With regard to in vitro embryo production, analysis of the methylation status of candidate imprints in IVM bovine and human oocytes revealed no or only marginal effects [20, 29, 30]. This data concurred with earlier findings in IVM-derived murine offspring, which showed that life span and most physiological and behavioural parameters were not impacted by IVM [31]. In contrast to the short exposure time to in vitro culture conditions that IVM entails, post fertilization IVC until blastocyst can last from 1 to 8 days (3–4 days in mice [28], 5–6 days in human [32] and 7–8 days in cattle [33]), therefore it is not surprising that it has been associated with impaired imprinting for several genes in murine blastocysts and placental murine tissues [16, 34, 35]. In cattle, several reports have been published detailing the impact of IVM, IVF and IVC on single, multiple or global gene expression patterns of bovine oocytes and embryos [4, 36–39]. Aberrant expression appears to persist beyond elongation and implantation [40, 41]. The divergent transcriptomic data is likely to be associated with altered epigenetic regulation [6]. Similarly, the high mortality rates and morphological anomalies observed in surviving cloned calves [42–44] are likely due to erroneous epigenetic reprogramming, as severe hypomethylation of imprint DMRs [45–49] in tissues recovered at various stages of development from day 17 to full term has been reported. Most recently, analysis of kidney, brain, muscle, and liver of ART-derived (produced in vitro) day ~ 105 large offspring syndrome (LOS) fetuses revealed dysregulation of imprinted gene expression, with the number of misregulated genes positively correlated with an increasing magnitude of overgrowth in LOS fetuses. DNA methylation analysis in these fetuses at the DMR of three imprinted genes, *SNRPN*, *NNAT*, and *PLAGL1*, also revealed some tissue specific aberrant methylation patterns [50].

**Fig. 1:**
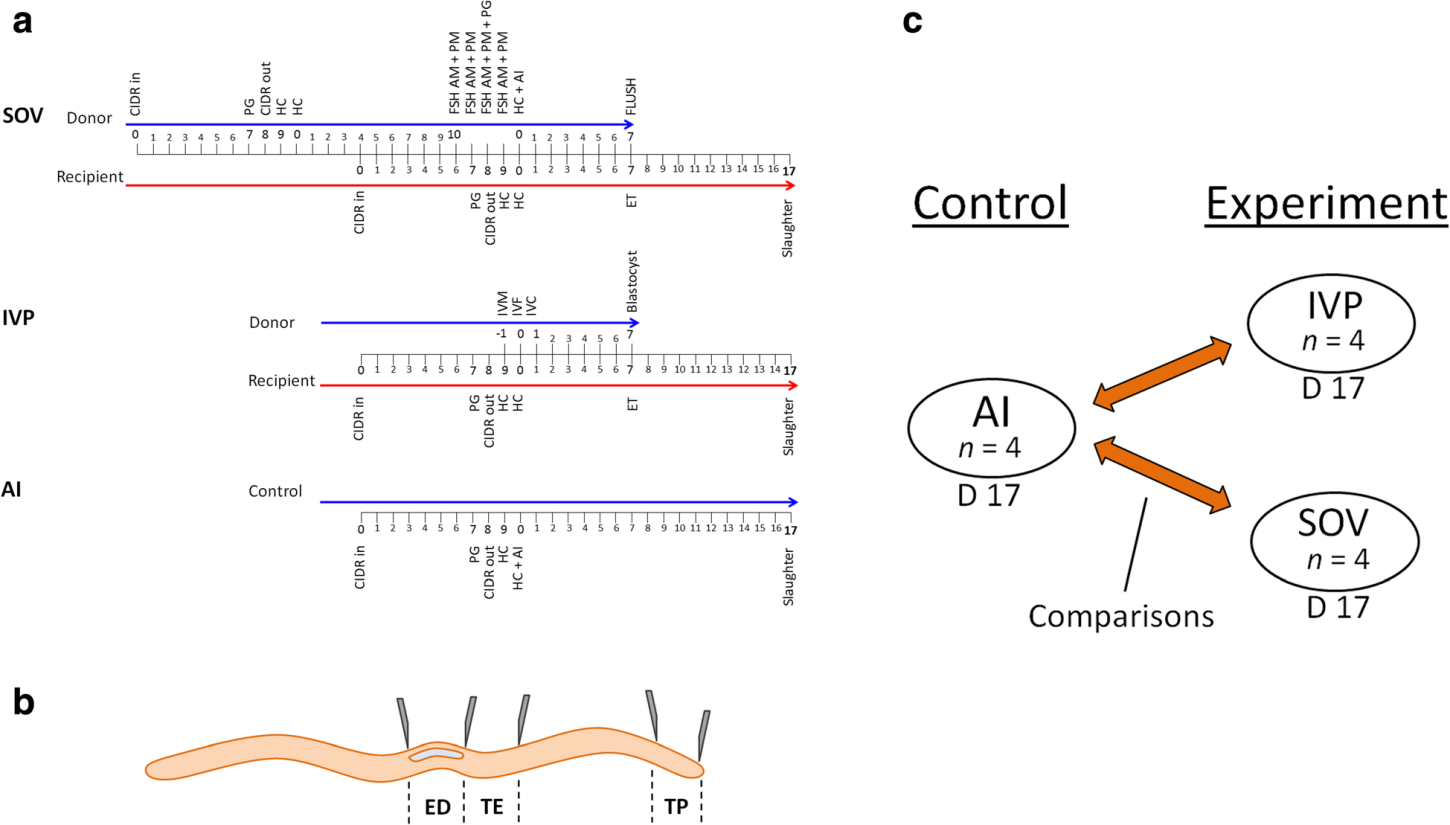
Experimental overview. **a** Synchronization protocols used to generate day 17 in vivo conceptuses (AI) and day 17 conceptuses derived from assisted reproduction technologies (SOV and IVP). **b** Schematic representation of the micro-dissected embryonic regions used in this study. **c** Overview of the multiple comparisons performed using the EDMA platform. ED = embryonic disc, TP = trophectoderm peripheral, TE = trophectoderm adjacent to embryonic disc. CIDR, Controlled internal drug release, PG, prostaglandin F2 alpha injection, HC, heat check, FSH, follicle stimulating hormone, AI, artificial insemination, ET, embryo transfer, IVM, in vitro maturation, IVF, in vitro fertilisation and IVC, in vitro culture

Advances in genome-wide methylation analyses offer the opportunity to assess the effect of routine ART protocols on the global epigenetic landscape of gametes and embryos. Recently, the EmbryoGENE network at the University Laval, Quebec (http://emb-bioinfo.fsaa.ulaval.ca/) developed a microarray based methylation analysis platform for assessing genome wide methylation patterns using small quantities of DNA from bovine embryos [51]. This technology has been used to (1) demonstrate a link between S-adenosyl methionine supplementation, from the 8 cell stage until blastocyst, and DNA methylation in resultant blastocysts [52], (2) analyze the impact of different in vitro embryo culture lengths on DNA methylation of transferred embryos [53], (3) elucidate the effect of fatty acid exposure during oocyte maturation and embryo culture on blastocyst DNA methylation [54] and (4) identify differentially methylated loci in spermatozoa of monozygotic twin bulls [55]. Using this technology we evaluated the effect of oocyte maturation, fertilization and embryo development under in vitro (IVP) conditions*,* and the effect of ovarian hyperstimulation (SOV). Embryos were developed under these two conditions, separately, until day 7 (blastocyst stage) then transferred singly to recipient animals for recovery at day 17 (peri-implantation) for DNA methylation analysis. All IVP and SOV conceptuses were compared to the DNA methylation profiles of single ovulation in vivo conceptuses from non-stimulated synchronised animals (AI) (Fig. 1). Four differentially methylated gene bodies, identified on the EDMA array, were analyzed by pyrosequencing. Additionally, targeted analysis of DNA methylation at one paternally methylated (*H19*) and five maternally methylated (*SNRPN*, *PLAGL1*, *PEG10*, *IGF2R*, *MEST*) imprinted loci was also carried out in all IVP, SOV-derived and AI embryo samples. Ingenuity Pathway and gene expression analyses were performed to assess the functional implications of ART-induced differential DNA methylation.

## Results

### Total significantly differentially methylated loci associated with SOV and IVP

**Fig. 2:**
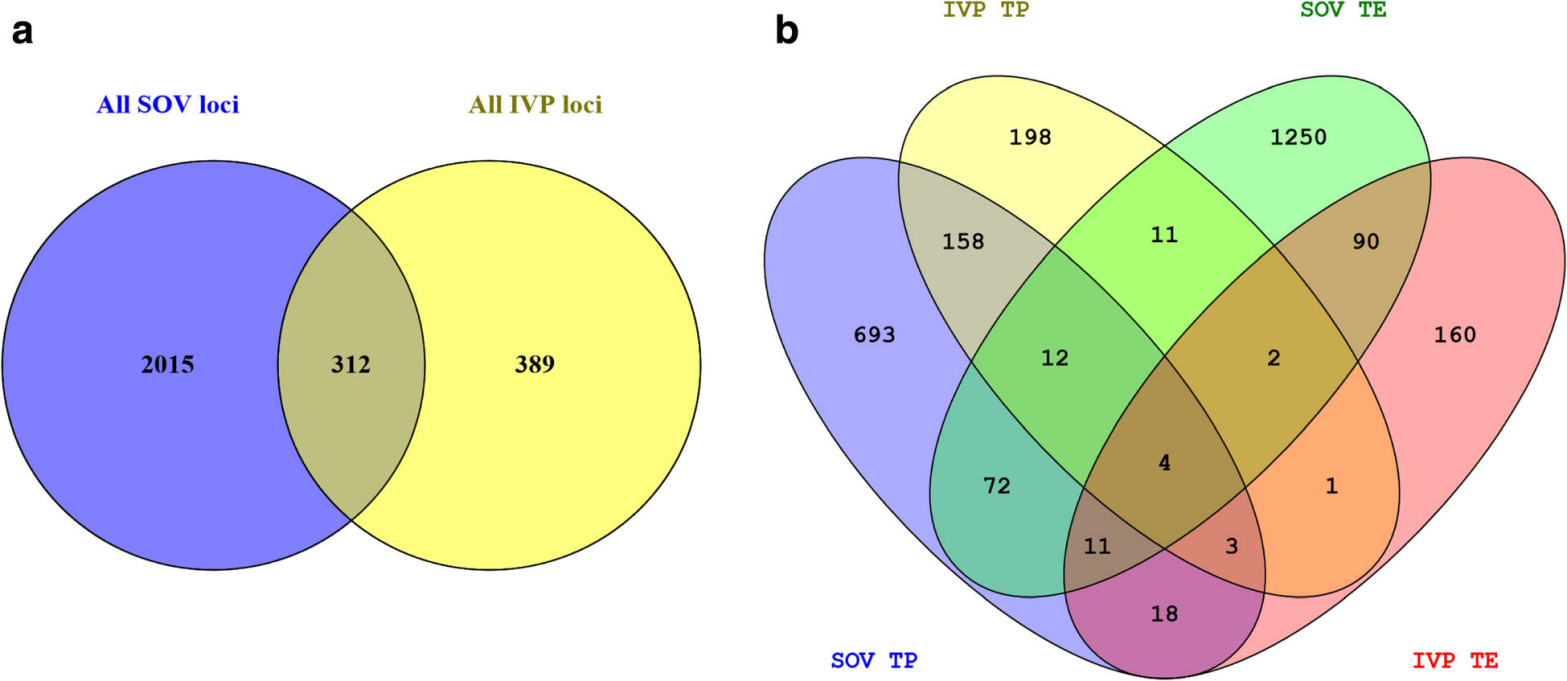
**a** 2-way venn diagram representing overlap of differentially methylated loci between SOV and IVP conceptuses. Duplicate probes that were identified in multiple groups were removed, therefore the total number of loci for SOV and IVP is less than detailed in Table 1, **b** 4-way venn diagram showing overlap of significant probes for the TE and TP tissues. Images generated using Venny http://bioinfogp.cnb.csic.es/tools/venny/index.html)

8134 loci were differentially methylated between AI and SOV-derived and IVP conceptuses. Taking a high stringency approach, only sequences with hybridization to both sense and matching anti-sense probes were analyzed (total 47,110 loci) and only those probes where both the sense and anti-sense probe achieved significance (*P* ≤ 0.05) and reached the fold-change threshold (≥ 1.5) were considered to be differentially methylated. Thus only loci that had overlapping probes yielding the same signal i.e. loss or gain of methylation relative to control samples were recorded as differentially methylated; 3140 loci met these criteria (Table 1). Overall, SOV and IVP resulted in an increased number of loci that were more methylated than the control conceptuses, 67.7% of the loci had increased methylation whereas only 32.3% of the loci had lower levels of methylation than control AI conceptuses. To determine if either SOV or IVP regimes had a different impact on the methylation of resultant conceptuses the total number of differentially methylated loci from each treatment was investigated. The effect of treatment on the number of differentially methylated loci was much more pronounced in conceptuses generated by SOV (77.3%) than those using in vitro techniques (22.7%). Fewer than 10% (312 loci) were consistent between SOV and IVP conceptuses (Fig. 2a). Analysis of methylation changes across three embryonic regions (ED, TE & TP) in all IVP and SOV conceptuses revealed that the majority of methylation changes were occurring in the trophectoderm (ED = 1.2% vs TE = 55.4% and TP = 43.3%). The full list of probes and their genomic coordinates are outlined in Additional file 1.

**Table 1:** Total number of differentially methylated probes

Region	Treatment	Up vs AI	Down vs AI	Total
ED	SOV	1	3	4
	IVP	6	29	35
TE	SOV	1316	136	1452
	IVP	164	125	289
TP	SOV	397	574	971
	IVP	243	146	389
Total		2127	1013	3140

*ED* = embryonic disc, *TP* = trophectoderm peripheral, *TE* = trophectoderm adjacent to embryonic disc, *SOV* = superovulation-derived conceptus, *IVP* = in vitro-derived conceptus

### Embryo production specific effects on DNA methylation

**Fig. 3:**
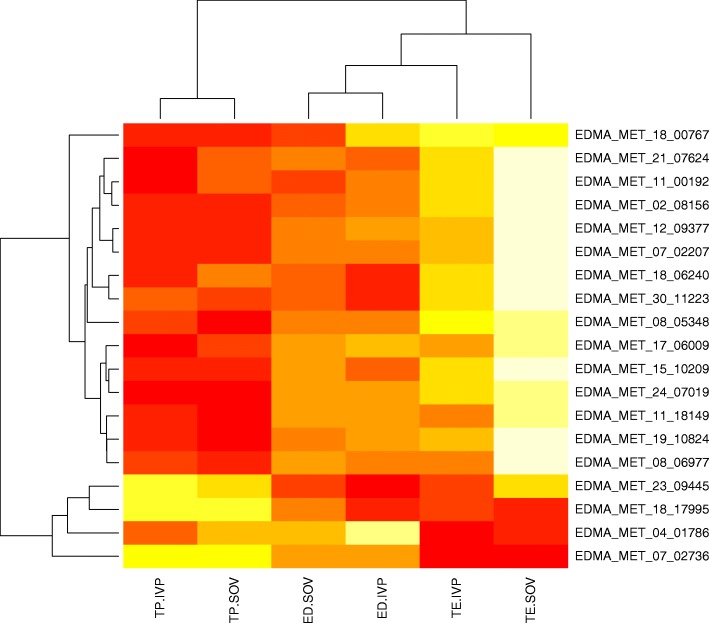
Heatmap of significant probes that exhibit differences in methylation state and direction in different tissues. A small number of loci show differences in the direction of change in methylation in different embryonic regions ED = embryonic disc, TP = trophectoderm peripheral, TE = trophectoderm adjacent to embryonic disc, SOV = superovulation-derived embryo, IVP = in vitro-derived embryo. The ID of each probe is outlined on the right hand side of the map and their genomic location can be found at http://emb-bioinfo.fsaa.ulaval.ca/bioinfo/html/index.html

The 3140 differentially methylated regions were queried to elucidate if there was any overlap between treatments or across embryonic regions. For this analysis the 39 loci significantly differentially methylated in the ED were omitted, as most of the significant differences were found in TE and TP tissue (3101 loci) comparisons. Significantly differentially methylated loci from IVP and SOV conceptuses were compared (Fig. 2b). The SOV conceptuses had 1250/1452 (86%) regions that were unique to TE and the IVP conceptuses had 160/289 (55%) regions that were unique to TE. For the TP samples 693/971 (72%) and 198/389 (51%) of the loci were differentially methylated following SOV or IVP, respectively. 90 regions in the TE and 158 in the TP regions were aberrantly methylated in both SOV and IVP conceptuses. There were 19 probes (y-axis, Fig. 3) that were significant in more than one contrast and showed changes in the direction of the effect. Most of the changes occurred between TE and TP contrasts, demonstrating that differential methylation direction can vary across both trophectoderm tissues.

### Underlying sequence features of differentially methylated loci

**Fig. 4:**
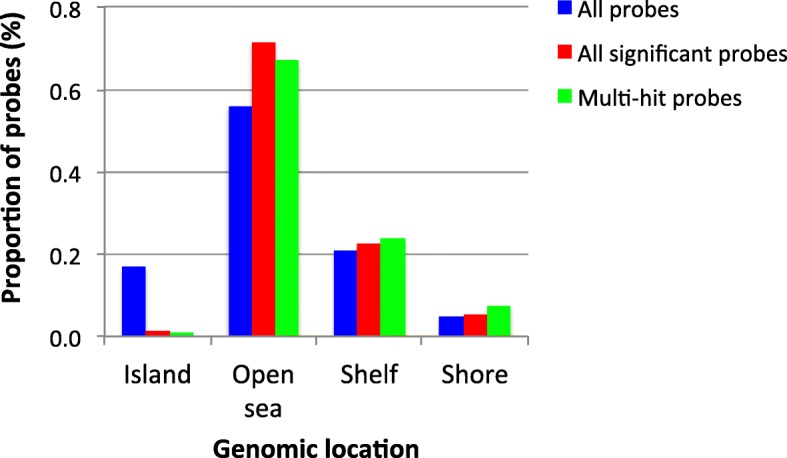
Distribution of differentially methylated loci. Breakdown of the percentage of differentially methylated loci in each genomic location (CpG islands, Open sea, Shelf and Shore), where both sense and anti-sense probes were significant in at least one contrast. The parameters used to define CpG Island, Open Sea, Shelf and Shore are outlined in [51]

Following the identification of sense-antisense probes that had statistically significant differences in methylation (*n* = 3140), their distribution across the genome was determined. As predicted from human array studies [56], the proportion of differentially methylated loci mapping within CpG islands was low, 36/3140 = 1.1% (Fig. 4 and Table 2). Given that we and others have shown that gene body methylation can facilitate transcription [57–60], the number of significant probes that were located within intragenic regions (coding and non-coding regions within the transcribed sequence) was calculated (Table 2). Irrespective of production method (IVP or SOV), 968 of the 3140 probes mapped to intragenic regions (30.8%), only a very small proportion (13/968; 1.3%) were found in the ED, with the remaining probes being split between TP (445/968; 46%) and TE (510/968; 52.7%). The data was also mined to identify whether DNA methylation aberrancies were occurring at loci encoding microRNAs, molecules that are involved with post-transcriptional gene regulation [61]. Disrupted DNA methylation was detectable at a single miRNA (miRNA 2890), in the peripheral trophectoderm (TP) of superovulated conceptuses.

**Table 2:** Differentially methylated probes mapping to gene bodies and CpG islands

	ED SOV	TE SOV	TP SOV	ED IVP	TE IVP	TP IVP
Total	4	1452	971	35	289	389
CpG Island	0	18	7	0	3	8
% in CpG Island	0	1.2	0.7	0	1.04	2.1
Gene Body	2	432	309	11	78	136
% in Gene Body	50	29.8	31.8	31.4	27	35
Gene body up	0	393 (91%)	132 (43%)	0	35 (45%)	85 (62.5% )
Gene body down	2 (100%)	39 (9%)	177 (57%)	11 (100%)	43 (55%)	51 (37.5%)

*ED* = embryonic disc, *TP* = trophectoderm peripheral, *TE* = trophectoderm adjacent to embryonic disc, *SOV* = superovulationderived embryo, *IVP* = in vitro-derived embryo

### Under-representation of differential methylation at CTCF loci

The number of significant probes that were located in CTCF recognition sites was determined using computationally predicted CCCTC-binding factor (CTCF) sites and their coordinates transferred to the bosTau6 (UMD3.1) assembly, using the LiftOver tool from UCSC. A total number of 7 of the 3140 differentially methylated fragments were located within the predicted CTCF binding sites. This compares to 746 of the 48,530 CTCF recognition sites in the total set of significant fragments that were analyzed. This means we found a 7.5-fold under-representation of CTCF sites in the differentially methylated loci (0.2% in differentially methylated fragments vs 1.5% in all fragments), which was highly significant (*p* < 1.3e-09 by Proportional Test).

### Array validation

**Fig. 5:**
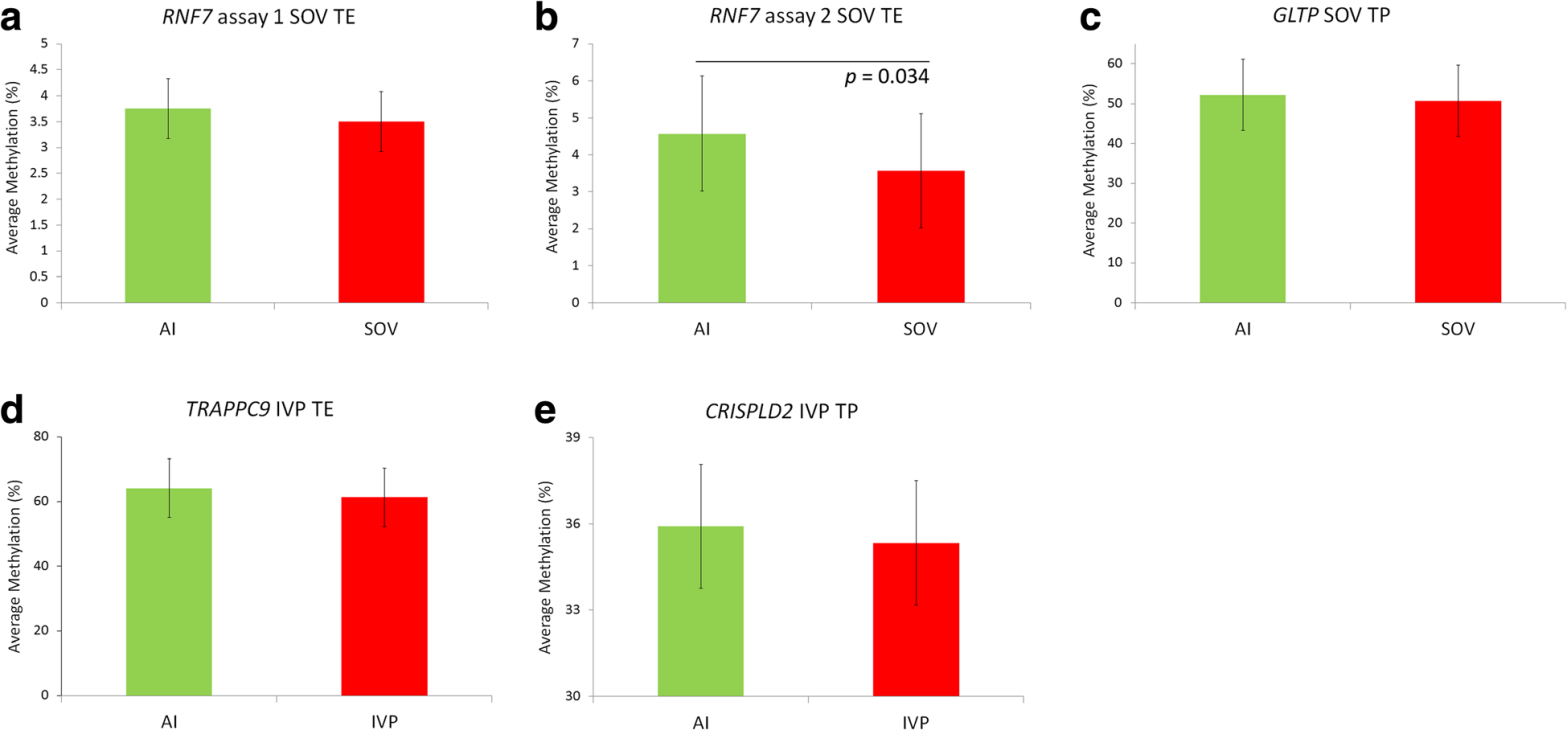
Pyrosequencing analysis of genes with differentially methylated gene bodies. Four genes identified as having differentially methylated intragenic regions by the EDMA analysis were selected for pyrosequencing. All assays confirmed the directionality of the change in methylation at these loci between control samples and ART samples (**a**–**e**). DNA methylation was significantly lower in day 17 SOV TE samples, relative to in vivo controls (**b**).

The EDMA has been validated previously by pyrosequencing analysis of DNA isolated from sperm and blastocyst samples [51]. In this study, DNA methylation was further analysed at four loci identified as being differentially methylated on the EDMA platform. Pyrosequencing assays were located within the intragenic regions of *RNF7* (*RNF7* has two assays covering separate CpGs – *RNF7* assay 1 and *RNF7* assay 2), *GLTP*, *TRAPPC9* and *CRISPLD2*. These loci were selected based on their fold-change, *P-*values and that the representative array probes contain at least one enzyme restriction site specific to the enzymes used for methyl-sensitive digestion during sample preparation for the array. They also represent comparisons of the following samples; SOV TE v AI TE, SOV TP v AI TP, IVP TE v AI TE and IVP TP v AI TP. Pyrosequencing confirmed the direction of methylation changes at these loci (loss of methylation at each locus), with *RNF7* assay 2 reaching significance (*P* ≤ 0.05) (Fig. 5).

### DNA methylation analysis of imprinted genes

**Fig. 6:**
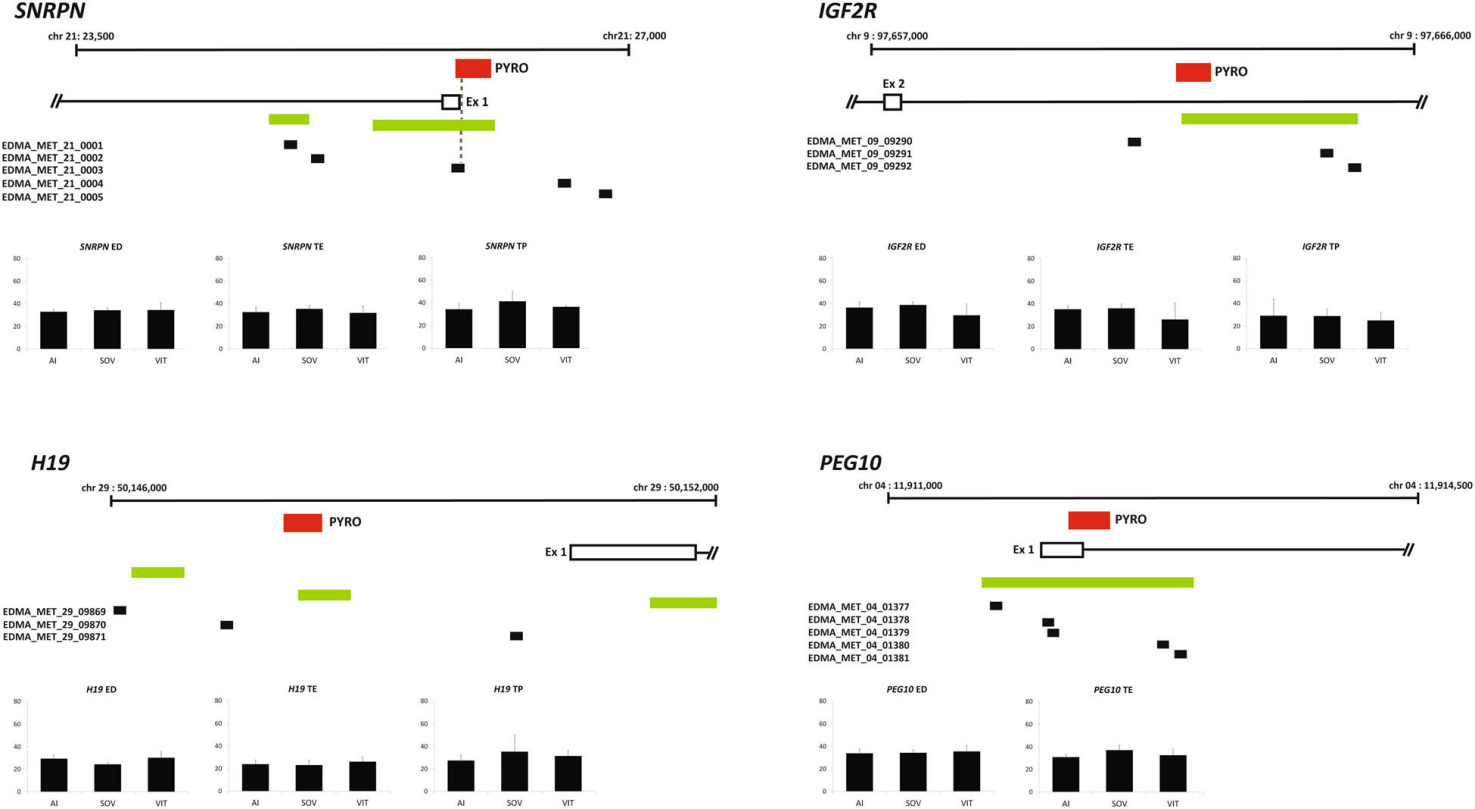
Location of EDMA probes and pyrosequencing assays at imprinted DMR loci that showed no ART-induced differential methylation. Loci analysed by pyrosequencing are labelled in red. The location of EDMA probes are indicated by black segments and CpG islands are green

**Fig. 7:**
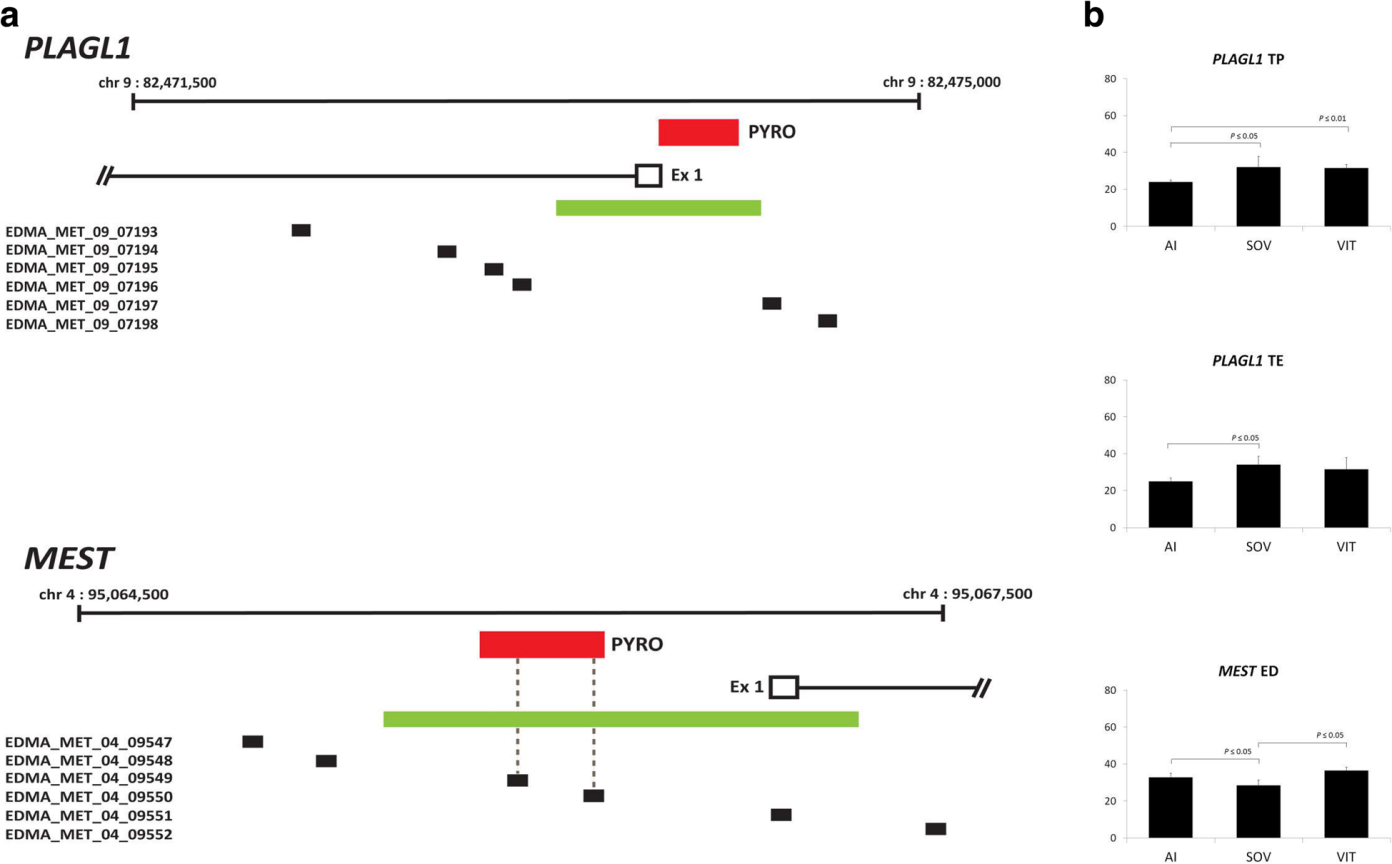
**a** Location of EDMA probes and imprinted DMR loci analysed by pyrosequencing. Loci analysed by pyrosequencing are labelled in red. The location of EDMA probes are indicated by black segments and CpG islands are green. Probe positions and sequences analysed using pyrosequencing were mapped using the Embryogene UCSC genome browser and schematics designed using Adobe Illustrator. **b** DNA methylation of the MEST and PLAGL1 DMRs in control and ART conceptuses. The y-axis is average methylation (%). The number of CpGs analysed for each DMR has been outlined previously [71]

The methylation status at six imprinted gene DMRs (*SNRPN*, *PLAGL1*, *PEG10*, *IGF2R*, *MEST* and *H19*) was determined by selective mining of the array output for probes located at imprinted loci (probe locations are outlined in Fig. 6 and Fig. 7a). None of the probes that mapped to imprinted genes were differentially methylated in the EDMA platform (adjusted *P*-value ≥0.05). In a parallel experiment, pyrosequencing of the six imprinted genes was carried out. In general, the pyrosequencing results concurred with the array data, i.e. no significant differences (Fig. 6). However, the *PLAGL1* and *MEST* DMRs showed some significant sites (Fig. 7b). The *PLAGL1* DMR was differentially methylated in trophectoderm tissue from both SOV and IVP samples. CpGs at this locus were significantly more methylated in the TE (AI: 24.9% vs. SOV: 34% and IVP: 31.5%) and TP (AI: 23.9% vs. SOV: 32.1% and IVP: 31.5%) regions of day 17 ART-derived conceptuses, relative to AI conceptuses. Methylation at *MEST* was significantly lower in the ED of SOV samples when compared to both AI and IVP. Additionally, to identify any further putative imprinted genes that were differentially methylated in the current study we compared the aberrantly methylated loci from the array data with a previously published list of 105 genes known to be imprinted in human and mouse [50]. This revealed that five genes (*DDC*, *DHCR7*, *SFMBT2*, *TCEB3* and *TRAPPC9*) were overlapping between the significantly differentially methylated genes identified on the array and the previously published reference list of known mammalian imprinted genes (Additional file 2).

### Functional implications of ART induced DNA methylation alterations

To determine the potential impact of SOV and IVP-induced differential methylation, observed in this study, Ingenuity Pathway Analysis (IPA) and qPCR were performed to interrogate genes that had differential methylation confined within gene bodies of TE and TP samples. IPA results showed that genes populated categories including embryonic development, cellular development, tissue development, gene expression and organismal development; the top 7 ranked categories are presented in Table 3 and the complete IPA output is included in Additional file 3. Gene expression analysis identified a link between the loss of methylation at the *TCEB3* locus, observed in SOV TE and IVP TE samples (Additional file 1), and down regulation of *TCEB3* expression in SOV TE samples (*P* = 0.04732). qPCR results are summarized in Table 4.

**Table 3:** Gene Ontology analysis of genes with differentially methylated gene bodies

Rank	Category	Number of genes	*P*-value
1	Cancer	391	6.11 × 10–09
2	Molecular Transport	148	4.84 × 10–08
3	Cellular Assembly and Organization	148	1.01 × 10–07
4	Cellular Function and Maintenance	195	1.01 × 10–07
5	Cell Morphology	168	3.08 × 10–07
6	Organismal Development	141	1.06 × 10–05
7	Cell Death and Survival	218	1.10 × 10–05

**Table 4:** qPCR analysis of imprinted genes and genes with ART-induced gene body methylation aberrancies

Gene symbol (Chromosome)	Differential Methylation EDMA	NCBI Ref Seq ID	SOV TE	SOV TP	IVP TE	IVP TP
*TCEB3* (chr 2)	Gene body SOV TE and IVP TE	NM_001102333.1	***0.047*** **↓**	0.34	0.19	0.51
*OCRL* (chr X)	Gene body TE IVP and TE SOV	NM_001102191.2	**0.08**	0.25	0.12	0.28
*ATP1A1* (chr 3)	Gene body TP SOV and TP IVP	NM_001076798.1	0.70	0.67	0.92	0.89
*SNRPN* (chr 21)	N/A	NM_001079797.1	0.63	0.51	0.15	0.16
*H19* (chr 29)	N/A	NR_003958.2	0.62	0.32	0.88	0.45

[*P*-values are given, significant values (*p* ≤ 0.05 unpaired, two tailed t-test) are underlined in bold]

The downwards arrow represents that the gene is downregulated compared to control (AI)

## Discussion

Here we advance the field by comparing, separately, the impact of hormonal and in vitro manipulations of bovine gametes and early embryos on the DNA methylation of preimplantation conceptuses. This unique approach to studying the impact of these procedures on embryonic DNA methylation was performed using DNA from multiple embryonic regions of single conceptuses and compared to control DNA isolated from in vivo-derived conceptuses. Results from the current study provide evidence that both of these techniques are potentially altering genomic methylation patterns, compared to unstimulated in vivo control samples, but especially SOV.

Classically, DNA methylation has often been defined as a repressive genome modification associated with silencing gene expression [62, 63]. A number of studies have demonstrated that non-promoter DNA methylation (e.g. gene bodies and regulatory elements) may have an active role in regulating gene expression [57, 58, 64]. In this investigation a large proportion of the differentially methylated loci (26.3–50%) were located within gene bodies and the direction of methylation differences at four gene bodies, between control and SOV-derived or IVP-conceptuses, was confirmed by pyrosequencing. We and others have also recently shown that decreasing gene body methylation at such genes through use of methyltransferase-deficient systems results in *decreased* transcription, highlighting a *positive* role for methylation in the gene body in facilitating transcription [59, 60, 65]. This implies that the altered gene body methylation observed in our SOV-derived and IVP conceptuses could indeed have functional consequences. Furthermore, in silico functional analysis of all the differentially methylated loci, within gene bodies of SOV-derived and IVP conceptuses, confirmed that the associated genes populated biological relevant categories (embryonic development, cellular development, tissue development, gene expression and organismal development) for this stage of mammalian development. Results from our qPCR experiments confirmed a possible link between differential gene body methylation (detected by EDMA) and gene expression, at the *TCEB3* locus.

In the current study we also assessed the impact of SOV and IVP on DNA methylation at CpG islands and CTCF recognition sites. Both of these genomic features were underrepresented in loci that were differentially methylated following ART. Perturbations of DNA methylation at CGIs of tumor suppressor genes are characteristic of many cancers [57], while CTCF is fundamentally involved with connecting the gap between nuclear organization and gene expression, it also regulates several epigenetic processes, such as X chromosome inactivation, imprinting and non-coding RNA transcription [66, 67]. Therefore, given the functional importance of these genetic elements, two hypotheses emerge, either of which would account for the underrepresentation of these loci in the set of differentially-methylated loci we identified: (1) incurring DNA methylation changes above a threshold level at these regions could result in embryonic lethality or, (2) CGIs and CTCF binding sites are more resistant to SOV or IVP-induced methylation changes. However, validation of either hypothesis requires further investigation.

The almost complete absence of differentially methylated loci in the ED region compared to the TE and TP regions, might suggest that either the ED is protected from methylation perturbations, or that such perturbations in this region of the embryo result in mortality. In addition to the observation that the majority of differentially methylated loci were within the trophectoderm regions, there were also a small number of probes showing directional differences in methylation, depending on whether they were in the TE or TP. The observation that the majority of the methylation differences occurred in the trophectoderm is intriguing. During implantation the trophectoderm engages directly with the mother’s uterus giving rise to tissues of the placenta, creating an interface between mother and fetus that is essential for exchange of nutrients, gases, waste and maintenance of gestation [68]. These findings support the hypothesis in the literature that adverse pregnancy outcomes, following ART, arise from deficiencies in placental function [69].

As outlined earlier, the impact of ART on methylation and expression of imprinted genes remains divisive [12–22, 30, 50, 70]. For this reason, we investigated the methylation of six previously characterized DMRs; *IGF2R*, *PEG10*, *MEST*, *SNRPN*, *PLAGL1* and *H19* [71, 72] and found no differences in methylation on the array or by pyrosequencing. However, pyrosequencing did identify significant changes in methylation at both *PLAGL1* and *MEST* and *PLAGL1* is under-represented by array probes. For *PLAGL1* the lack of significant changes on the array is probably due to a lack of probes located within the DMR that was covered by pyrosequencing. The CGI spanning the *MEST* proximal promoter, first exon and part of the first intron was represented on the array by 3 probes, 2 of which directly overlapped the region analysed by pyrosequencing. The lack of a significant signal at these locations on the array could be, in part, due to the high stringency approach used to select significantly differentially methylated loci from the array or be due to a technical difference between array analysis and targeted analysis of methylation. This has been as discussed previously by others [73] and we have recently detailed the limitations of the EMDA platform [54]. In addition, although the EMDA platform is cost-effective, has a rapid turnaround time, a dedicated downstream analysis pipeline and has been specifically designed to assess DNA methylation patterns in bovine embryos using finite amounts of input DNA (1 – 10 ng) [51], it is not possible to get single nucleotide resolution maps of genome wide methylation patterns using this technology. This can be achieved using whole genome bisulfite sequencing (WGBS). Future investigations using this method will help to provide higher resolution profiles of DNA methylation in embryos generate using ART.

Five additional imprinted genes (*DDC*, *DHCR7*, *SFMBT2*, *TCEB3* and *TRAPPC9*), identified as imprinted in human and mouse [50], were identified as having aberrant gene body methylation in SOV (*DDC*, *DHCR7*, *SFMBT2*, *TCEB3* and *TRAPPC9*) and IVP (*TCEB3*) conceptuses here. This recently published study by Chen et al. identified aberrant methylation patterns and biallelic expression of imprinted genes in fetal organs of pregnancies following transfer of in vitro produced embryos. It was demonstrated that DNA methylation was perturbed at *PLAGL1*, *NNAT* and *SNRPN*. Furthermore, recent studies using the Illumina Infinium Human Methylation Array, pyrosequencing and qPCR to compare cord blood samples from ART and control pregnancies also revealed that the *PLAGL1* locus is sensitive to ART manipulations [74, 75]. The consensus between the current and earlier studies, that *PLAGL1* is sensitive to ART-induced methylation changes, is consistent with observations of an association between ART and patients with the human disorder Beckwith–Wiedemann syndrome [76, 77], thus highlighting *PLAGL1* as a key susceptibility marker to ART procedures.

## Conclusions

In summary, both IVP and SOV procedures were associated with genome wide differences in embryonic DNA methylation to different extents. Superovulation treatment was the major cause of differential methylation in this study. Changes to DNA methylation was region specific; the embryonic disc showing almost no alterations compared to a significant number of differences in trophectoderm tissues. The differentially methylated loci tended to cluster within intragenic regions, suggesting a non-random effect, and are enriched for cancer, cell morphology and development. There was also an effect of ART on DNA methylation at a small number of imprinted genes and gene expression at the *TCEB3* locus. Methylation differences at the *PLAGL1* locus were apparent by pyrosequencing; this is congruent with observations in the literature demonstrating the influence of ART on DNA methylation at imprinted loci. Overall this study provides evidence that ART induces alterations to the embryonic methylome, in addition, many of these alterations appear to occur in an ART intervention, tissue and gene -specific manner. The majority of which were observed in the trophectoderm of SOV-derived conceptuses. These experiments demonstrate that embryos developing from the zygotic stage to the blastocyst stage in a modified environment (in vitro culture conditions or oviduct microenvironment containing multiple SOV-derived embryos) and transferred to a ‘normal’ environment retain aberrant epigenetic programming. The observed ART-induced DNA methylation differences may lead to misregulation of gene expression later in development, reducing developmental potential and contributing, in part, to health complications such as fetal overgrowth and large-offspring syndrome (LOS). Indeed, a recent study using WGBS has shown a link between DNA methylation differences and the expression of a small number of genes in skeletal muscle recovered from day ~ 105 bovine LOS foetuses [78]. Our study bolsters the importance of a non-rodent model, particularly the cow, for providing comparative data for the human IVF and developmental programming fields and provides a base for future high-resolution Whole Genome Bisulfite Sequencing studies investigating the impact of ARTs on the embryonic genome in cattle.

## Methods

### Study design and number of comparisons

The experimental design for embryo production is illustrated in Fig. 1a. Four day 17 conceptuses of each type (4 x AI, 4 x IVP and 4 x SOV) that were fully intact upon flushing from the uterus were retained for experimental analysis. Each embryo was dissected into the following sections, as outlined in Fig. 1b; the embryonic disc (ED) and trophectoderm - embryonic disc adjacent (TE) and trophectoderm peripheral (TP). The entire embryonic disc was trimmed and for the TE and TP approximately 1 cm sections were isolated. The rationale to interrogate these regions separately was based on previous investigations demonstrating that differences, such as differences in morphology and function, occur between regions adjacent to the embryonic disc and the periphery of the trophectoderm. Multiple statistical contrasts (Fig. 1c), comprising four biological replicates of each type of embryo and each embryonic region (ED, TE and TP), were carried out using the 400 K EmbryoGENE DNA Methylation Array (EDMA http://emb-bioinfo.fsaa.ulaval.ca/). This bovine-specific array contains ~ 420,000 probes mapping to 359,738 loci, surveying 20,355 gene-regions and 34,379 CpG islands).

### Preparation of conceptuses

#### Animal synchronization and embryo collection

All animals were housed indoors in a slatted shed for the duration of the experiment and were fed a diet consisting of grass and maize silage supplemented with a standard beef ration. Cross-bred beef heifers (primarily Charolais beef heifers, or Simmental X Charolais and Limousin X Charolais crosses) were randomly assigned to be treated as unstimulated donors or recipients (i.e. single-ovulating, *n* = 20) or superstimulated donors (*n* = 9). Artificial insemination and IVF were carried out using frozen thawed semen from the same bull to limit any potential variability that may be introduced by using spermatozoa from multiple bulls. Animals were slaughtered at a local abattoir 17 days following insemination or 10 days subsequent to embryo transfer, using standard practice.

#### Unstimulated heifers

Collection of control in vivo-derived bovine conceptuses was performed using a previously described synchronization protocol [79], denoting these control conceptuses as ‘AI’ is based on a previous investigation [80]. Briefly, heifers (approximately 18–24 months old) were synchronized using an 8-day Controlled Internal Drug Release device (CIDR 1.36  g, Pfizer, Sandwich, Kent, UK) with administration of a prostaglandin F2α (PGF2α) analogue (2  ml Estrumate; Schering-Plough Animal Health, Hertfordshire, UK, equivalent to 0.5  mg cloprostenol) injection one day prior to removal of the CIDR. Animals were examined for estrus four times daily, from 36 h following PGF2α injection. Animals in standing estrus between 36 and 60 h were inseminated using frozen thawed semen. Reproductive tracts were recovered within 30 min of slaughter from animals on day 17 post insemination, and transported on ice. Conceptuses were recovered from reproductive tracts by flushing both uterine horns with 40 ml of PBS containing 5% fetal calf serum (FCS). All intact conceptuses were washed and dissected in PBS and then immediately snap frozen using liquid nitrogen.

#### Superstimulated donor heifers

Procedures for superstimulation were as described by Rizos et al. [81]. Beginning on day 10 of a synchronised oestrous cycle, heifers were superstimulated with a total of 455  IU FSH (13  ml Folltropin; Bioniche, Inverin, Galway, Ireland) given as twice daily intramuscular injections over 4 days on a decreasing dose schedule. Luteolysis was induced with 2  ml Estrumate (PGF2α) given on day 12 with the sixth injection of follicle stimulating hormone (FSH). All heifers received 2.5  ml Receptal (GNRH) at 40  h after PGF2α, the expected time of the luteinizing hormone (LH) surge [82]. Animals seen in standing estrus between 36 and 60 h were inseminated using frozen thawed semen. Inseminated animals were slaughtered and embryos recovered from reproductive tracts on day 7 and used for same day embryo transfer.

#### In vitro embryo production

The techniques for producing embryos in vitro have been described in detail previously [81], reagents were purchased from Sigma (Sigma-Aldrich, Ireland). Immature cumulus–oocyte complexes (COCs) were obtained by aspirating follicles from the ovaries of heifers and cows collected at killing. COCs were matured for 24  h in TCM-199 supplemented with 10% (*v*/v) FCS and 10  ng/ml epidermal growth factor at 39  °C under an atmosphere of 5% CO_2_ in air with maximum humidity. For IVF, matured COCs were inseminated with frozen-thawed Percoll-separated bull sperm at a concentration of 1 × 10^6^ spermatozoa/ml. Gametes were co-incubated at 39 °C under an atmosphere of 5% CO_2_ in air with maximum humidity. Semen from the same bull was used for all experiments. At ∼20  h post-insemination (hpi), presumptive zygotes were denuded and cultured in groups of 50 in 500  μl synthetic oviduct fluid media (SOF) supplemented with 5% FCS. Cleavage rate was recorded at 48 hpi and blastocyst development recorded at day 7 post-insemination (pi).

#### Unstimulated recipient heifers and embryo transfer

Control animals (AI) were oestrous synchronized, as described above, artificially inseminated on detection of estrus and slaughtered 17 days post insemination. Oestrous synchronised recipient animals were randomly assigned to receive a day 7 blastocyst stage embryo recovered from a stimulated heifer (SOV) or produced in vitro (IVP), 7 days following detection of estrus. Recipient animals were slaughtered 10 days post embryo transfer and conceptuses were recovered from reproductive tracts on day 17 of embryo development.

### Sample preparation

Day 17 conceptuses were processed for methylation array analysis by dissecting three embryonic regions from all control (AI) and treatment group samples (SOV and IVP) immediately after recovery from the reproductive tract. These regions were as follows: the embryonic disc (ED); the trophectoderm region directly adjacent to the embryonic disc (TE); and the peripheral tip of the elongated day 17 embryo (TP) (Fig. 1b). Genomic DNA and total RNA were isolated from single, dissected day 17 conceptuses using the AllPrep DNA/RNA Micro Kit (Qiagen, Manchester, UK) according to the manufacturers’ guidelines. DNA samples were quantified using a Qubit dsDNA HS assay kit (Invitrogen™, ThermoFisher Scientific Ltd., Dublin, Ireland). 10 ng of DNA from each sample was prepared for the array exactly as outlined in [51]. 100 ng total RNA from each region of all conceptuses and converted to cDNA as described previously [83].

### Microarray

For a complete outline of microarray design and probe locations see [51, 84] and the EmbryoGENE UCSC Genome Browser (http://emb-bioinfo.fsaa.ulaval.ca/bioinfo/html/index.html). A total number of 36 separate amplifications, comprising three regions (ED, TE, TP) from each of 12 conceptuses (4 × AI, 4 × SOV and 4 × IVP), were analysed in the present study. Quality control plots for all samples generated after EDMA microarray hybridization and data analysis are included in Additional file 4. The microarrays were processed using a custom pipeline outlined in [51]. The heatmap in Fig. 3 was generated in R using the heatmap function.

### EDMA data analysis

EDMA data was analysed as previously outlined [51] using the Limma package from Bioconductor [85, 86]. LOESS intra-array normalisation and quantile inter-array scale normalisations were performed. Normalised data was then fitted to a linear model and tested for differential methylation using Bayesian statistics. DNA methylation differences were considered significant when the *P* value was < 0.05 and the absolute log2 fold change threshold was ≥1.5. Eigen values were used to compare groups using the Bioconductor package MADE4 [87].

### Pyrosequencing and qPCR

Methylation analysis of six imprinted gene DMRs (*SNRPN*, *PLAGL1*, *PEG10*, *IGF2R*, *MEST* and *H19*) and four gene body regions identified as differentially methylated using the EDMA platform (*RNF7*, *GLTP*, *TRAPPC9* and *CRISPLD2*) was performed using pyrosequencing, as described previously [83, 88]. Briefly, a subset of tissue samples, collected as described above, were snap frozen, according to embryonic region, in 6 μl PBS and stored at − 80 °C. Prior to bisulfite PCR and pyrosequencing, samples were thawed, homogenised by vortexing for 1 min and 1 μl was removed for bisulfite modification of DNA using the EZ DNA methylation Direct kit, Zymo Research, USA. Modified DNA was eluted in 42 μl elution buffer (preheated to 50 °C) and 6 μl was used as template in PCR reactions. For PCR conditions and primer sequences see [71, 72]. *RNF7*, *GLTP*, *TRAPPC9* and *CRISPLD2* primers are outlined in Additional file 5: Table S1. Methylation values were used a continuous variables for statistical analysis. Sample group means for each gene were compared using ANOVA followed by post-hoc *t*-tests using a Tukey’s honest significant difference (HSD) multiple testing correction threshold of ≤0.05. For each gene, analysis of residual values (Q-Q plots and Anderson-Darling tests) showed that all data were normally distributed. All statistical analyses were performed using the Minitab version 16 software package (Minitab Inc., PA, USA). qPCR was carried out in 15 μl reactions containing 7.5 μl Fast Sybergreen mastermix (Applied Biosystems, Foster City, CA, USA), 0.3 μm of each primer and 5 μl of a 1/10 dilution of cDNA. Raw CT values were imported into qbase^plus^ (Biogazzelle, Zwijnaarde, Belgium) were data was calibrated, normalised and expression values (CNRQ) for each gene was determined. Target genes, *TCEB3*, *OCRL* and *ATP1A1*, were selected as they were shown to be differentially methylated in at least two comparisons on the array. *SNRPN* and *H19* were also included as they are two of the most extensively studied imprinted genes. Target genes were normalised using two stable reference genes, *H3F3A* and *GAPDH* (qPCR primers are listed in Additional file 5: Table S2). Statistical analysis for each gene (unpaired, two tailed t-tests) was carried out using the stat wizard function in qbase^plus^.

### IPA analyses

Ingenuity Systems Pathway Analysis (IPA; Ingenuity Systems, Redwood City, CA, USA) was used to identify canonical pathways and functional processes of biological importance within the lists of all differentially methylated regions that were located within gene bodies. Gene bodies were defined as all coding and non-coding regions within the transcribed sequence. Intensity on the array does not necessarily match methylation pattern or level for the complete gene-defined region. Functional analysis of differentially methylated loci, within gene bodies, was performed to characterize biological processes that could potentially be affected by ART. Right-tailed Fisher’s exact tests were used to calculate a *P*-value for each of the biological functions assigned to a list of differentially methylated gene bodies.

## Additional files

Additional file 1: Full list of differentially methylated probes and their genomic coordinates. (XLS 820 kb)

Additional file 2: Comparison of differentially methylated genes with previously published imprinted genes. (XLS 28 kb)

Additional file 3: IPA output. (XLS 72 kb)

Additional file 4: Quality control plots for all samples generated after EDMA microarray hybridization and data analysis. (PDF 2803 kb)

Additional file 5: **Table S1.** Pyrosequencing primers used for array validation. **Table S2.** Primers used for gene expression analysis. (DOCX 17 kb)

## Abbreviations

AI: Artificially inseminated
ART: Assisted reproductive technologies
CGI(s): CpG island(s)
CIDR: Controlled internal drug release device
COCs: Cumulus-oocyte complexes
CpG(s): Cytosine guanine dinucleotide
CTCF: CCCTC-Binding Factor (Zinc Finger Protein)
DMR(s): Differentially methylated region(s)
DNA: Deoxyribonucleic acid
ED: Embryonic disc
EDMA: EmbryoGENE DNA Methylation Array
FCS: Fetal calf serum
FSH: Follicle stimulating hormone
GnRH: Gonadotropin releasing hormone
hpi: hours post-insemination
ICSI: Intracytoplasmic sperm injection
IFC: In vitro follicle culture
IPA: Ingenuity pathway analysis
IVF: In vitro fertilisation
IVM: In vitro maturation
IVP: In vitro produced
LH: Leutinizing hormone
LOS: Large offspring syndrome
PBS: Phosphate buffered saline
PGF2α: Prostaglandin F2α
qPCR: quantitative real time PCR
SOF: Synthetic oviduct fluid
SOV: Superovulation-derived
TCM-199: Tissue culture media 199
TE: Trophectoderm adjacent to the embryonic disc
TP: Peripheral trophectoderm
UCSC: University of California, Santa Cruz
