# Supplementary material for: Intragenic sequences in the trophectoderm harbour the greatest proportion of methylation errors in day 17 bovine conceptuses generated using assisted reproductive technologies
Source: BMC Genomics. 2018 Jun 5;19:438. doi: 10.1186/s12864-018-4818-3 (PMC5987443; doi:10.1186/s12864-018-4818-3)
Supplement: Supplementary file 4 — Quality control plots for all samples generated after EDMA microarray hybridization and data analysis. (PDF 2803 kb) [file 12864_2018_4818_MOESM4_ESM.pdf]

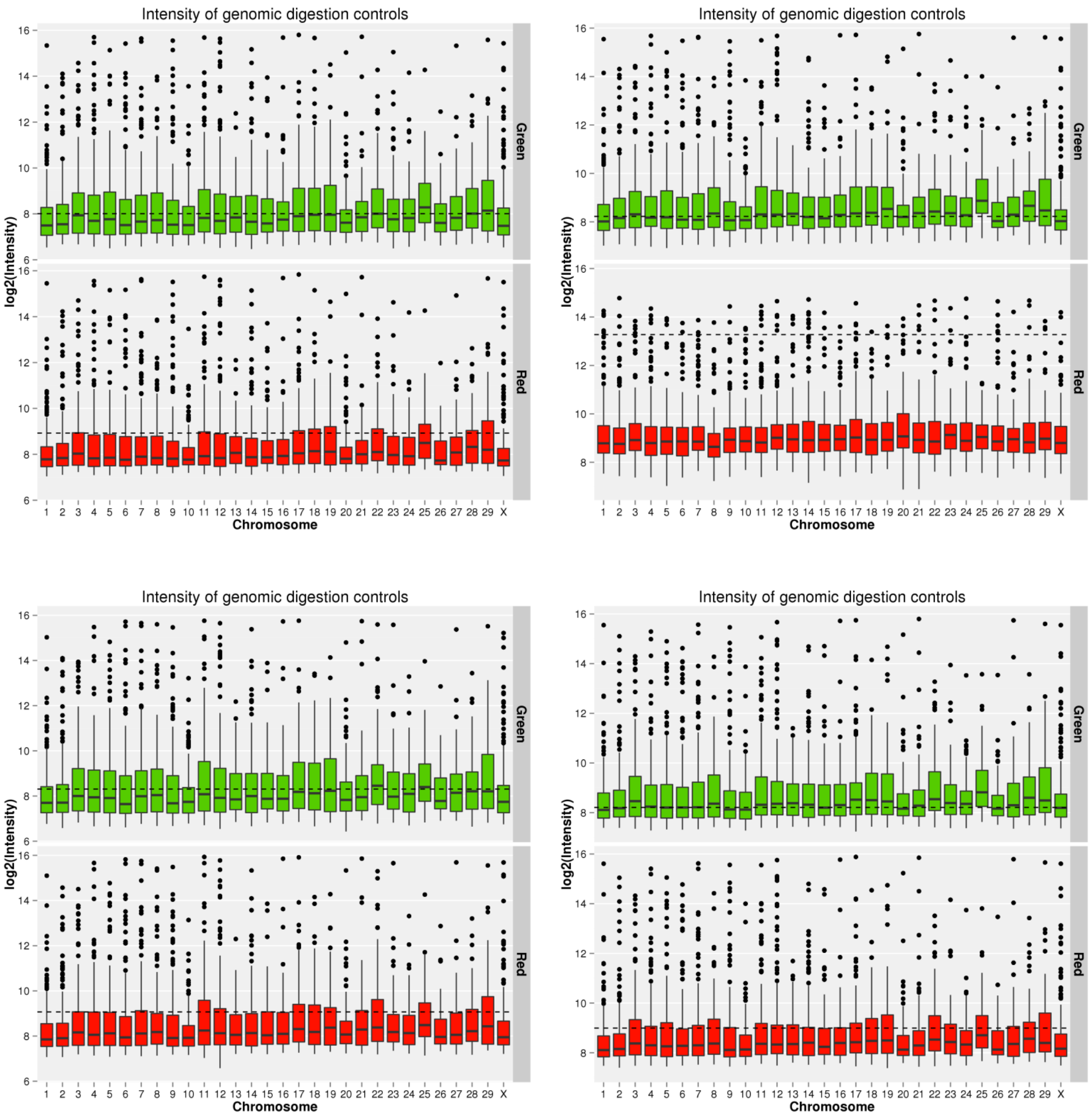

IVP and VIVO genomic DNA digestion controls (ED).

Signal intensity of control probes designed to overlap an MseI site. Probes corresponded to loci distributed throughout the bovine genome. The dashed horizontal line represents the limit of detection (mean intensity of negative controls plus four standard deviations). Intensities below this line indicate successful genomic fragmentation by *MseI*.

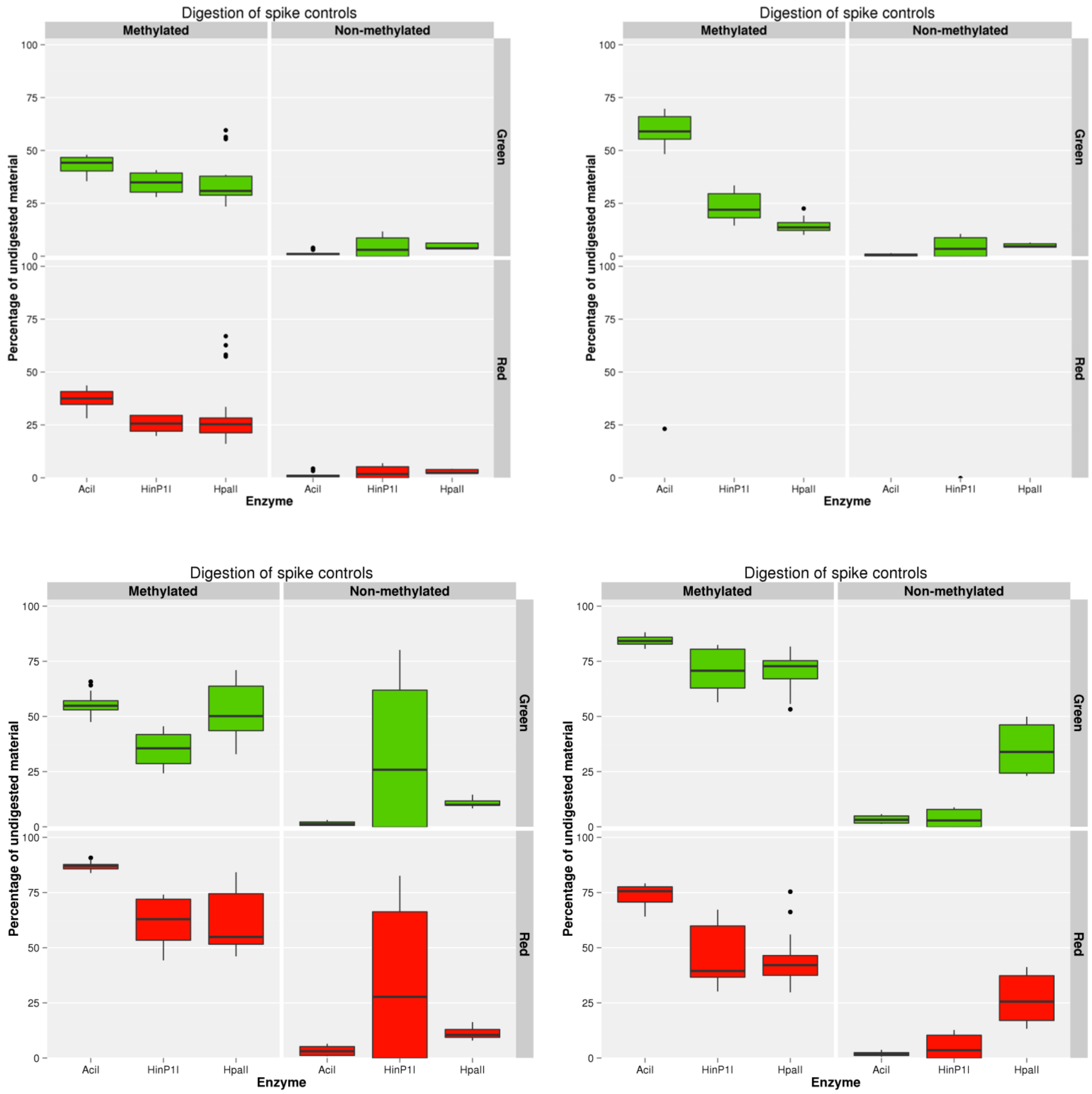

IVP ED spiked-in-control DNA digestion.

Digestion of synthetic spiked-in control DNA pairs by methyl-sensitive restriction enzymes, based on microarray signal. Signals from unprotected (unmethylated) fragments (right) are near background values.

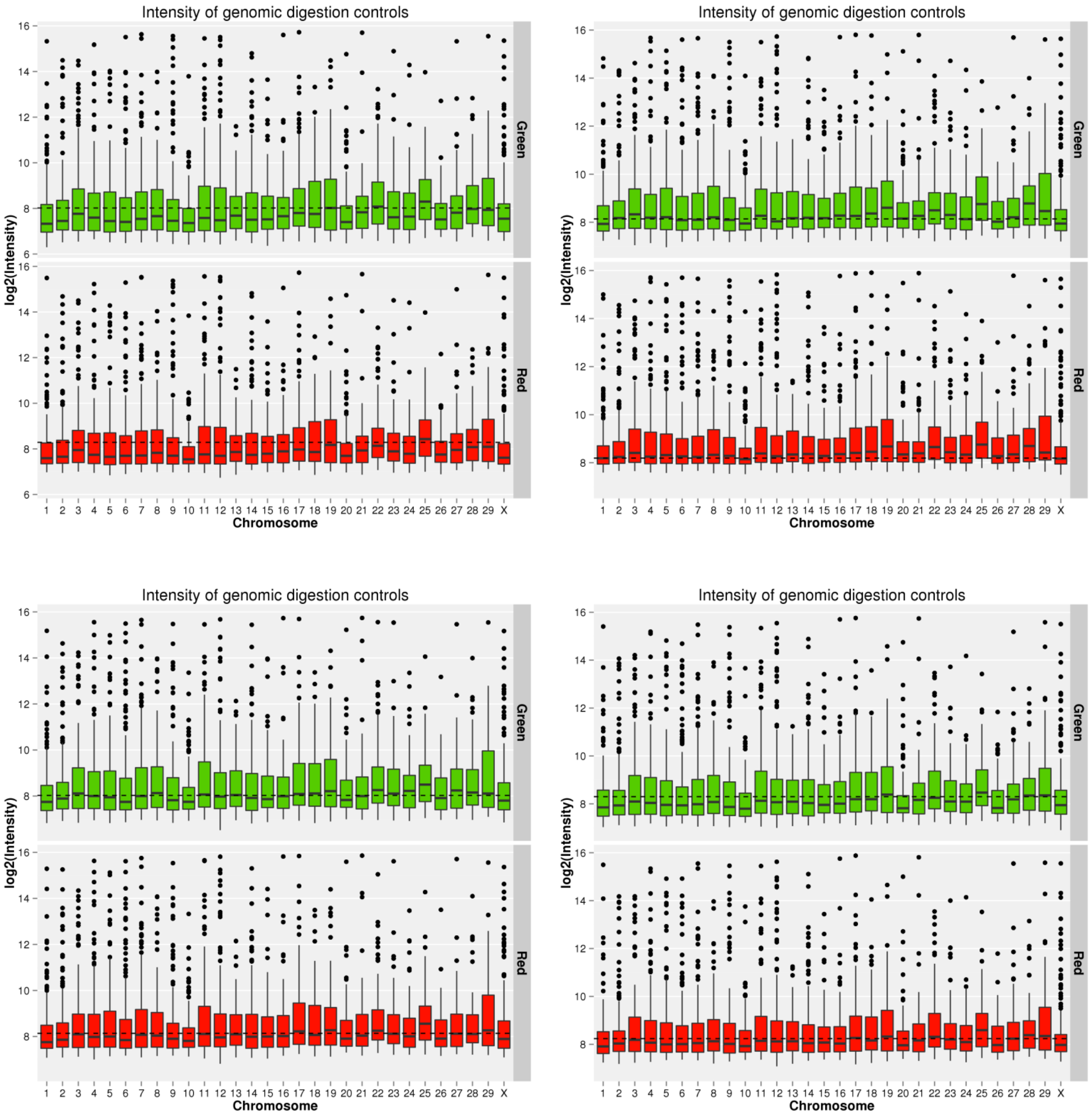

SOV and VIVO genomic DNA digestion controls (ED).

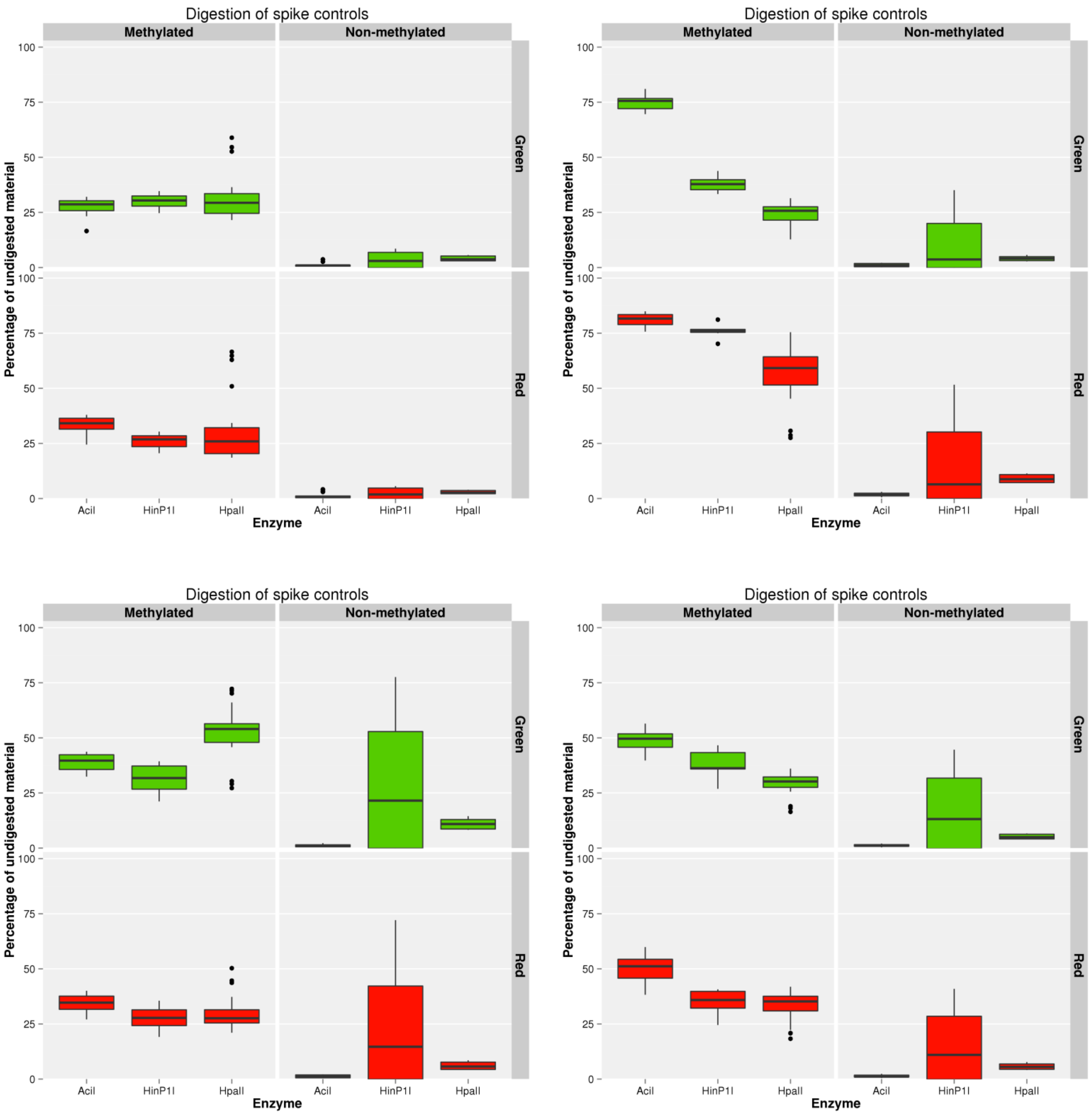

SOV ED spiked-in-control DNA digestion.

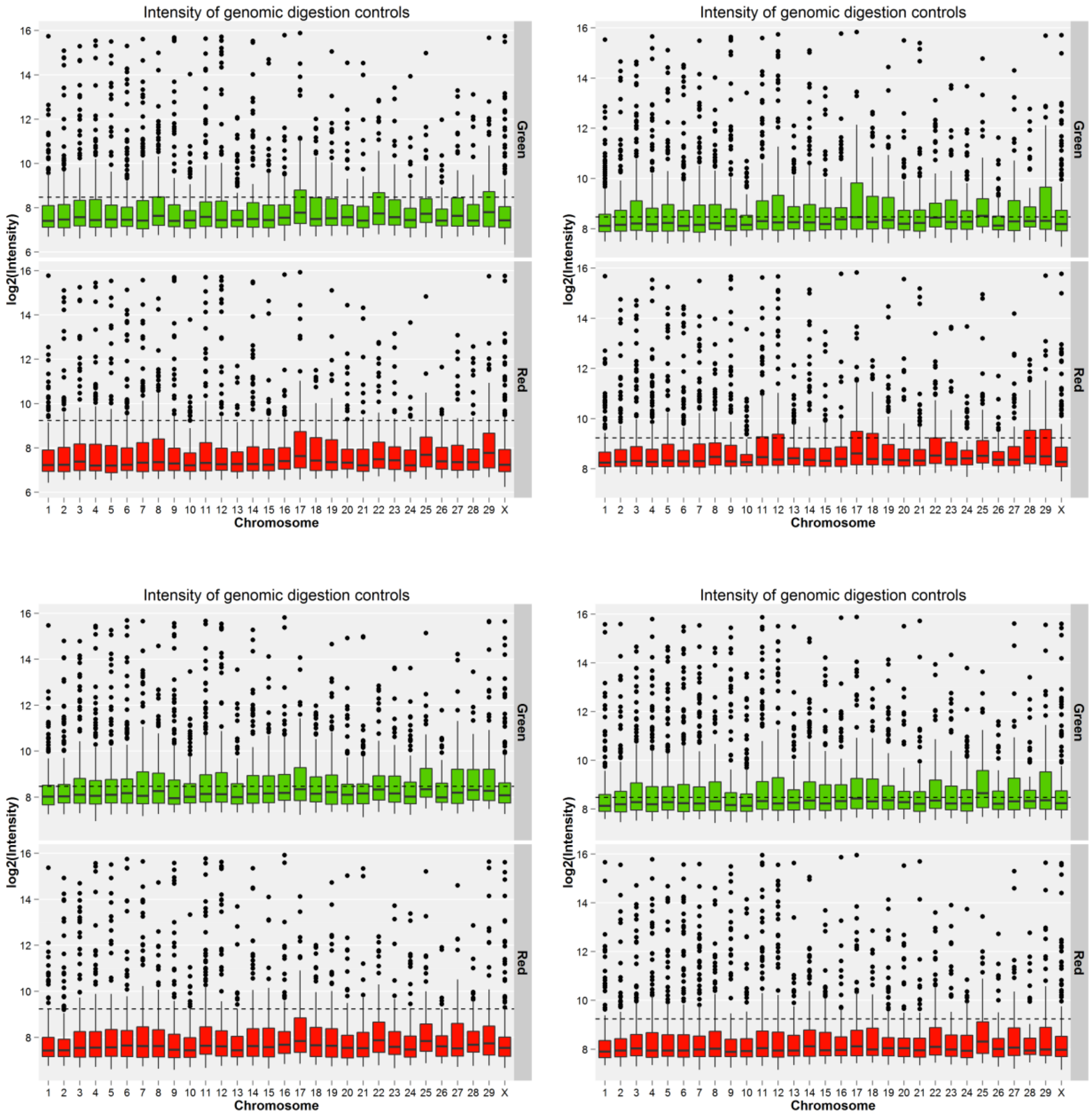

IVP and VIVO genomic DNA digestion controls (TE).

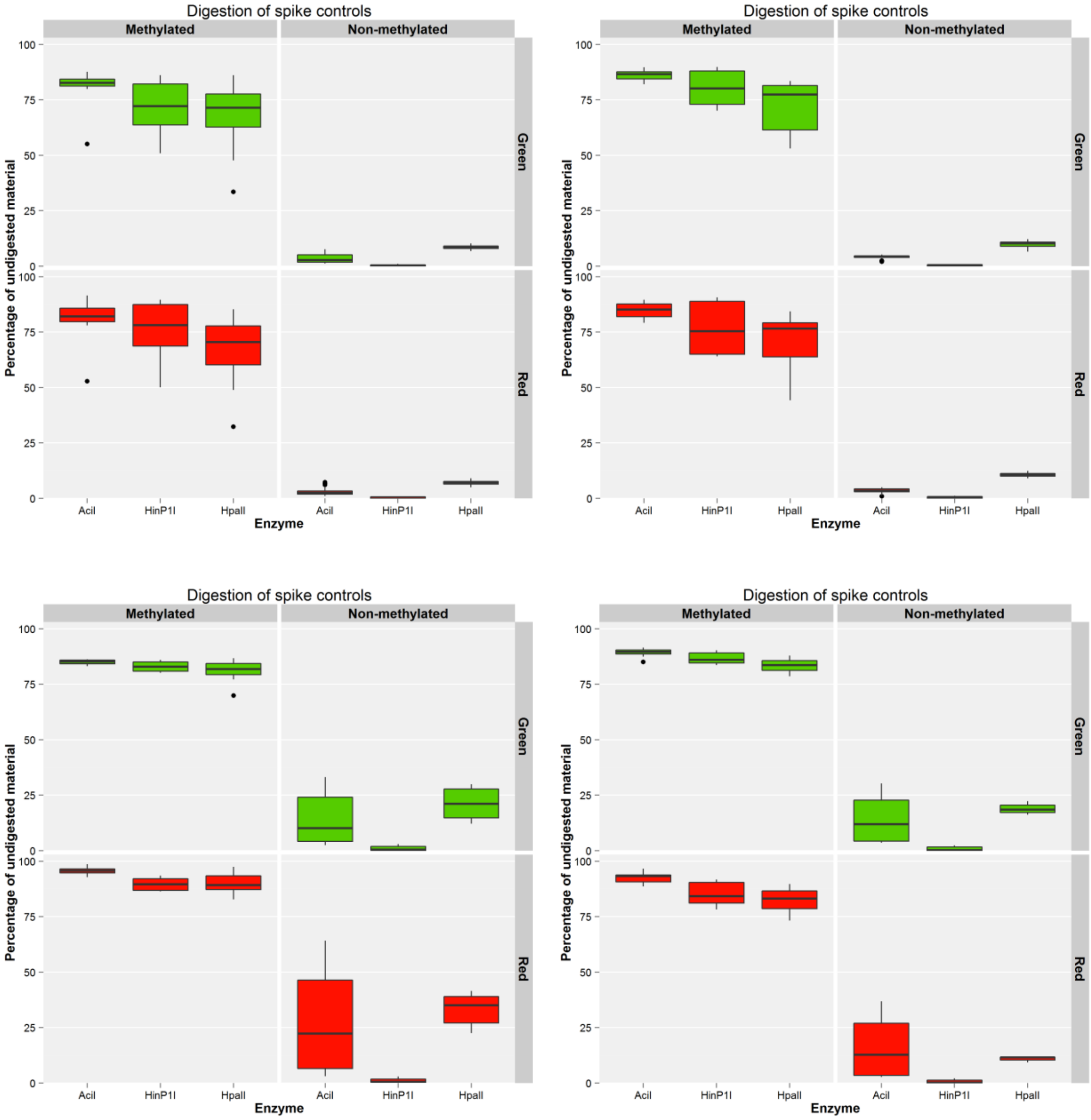

IVP TE spiked-in-control DNA digestion.

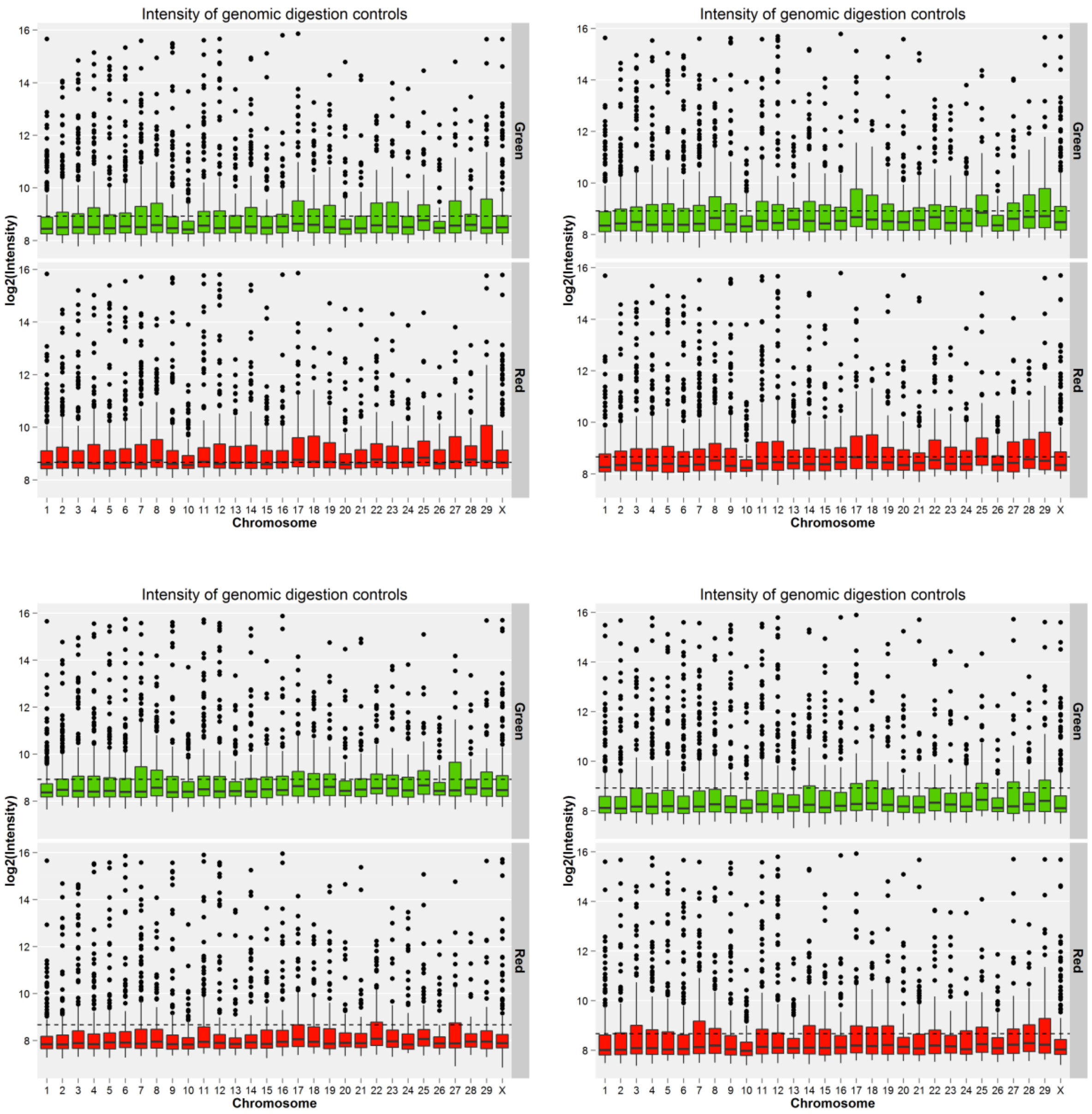

SOV and VIVO genomic DNA digestion controls (TE).

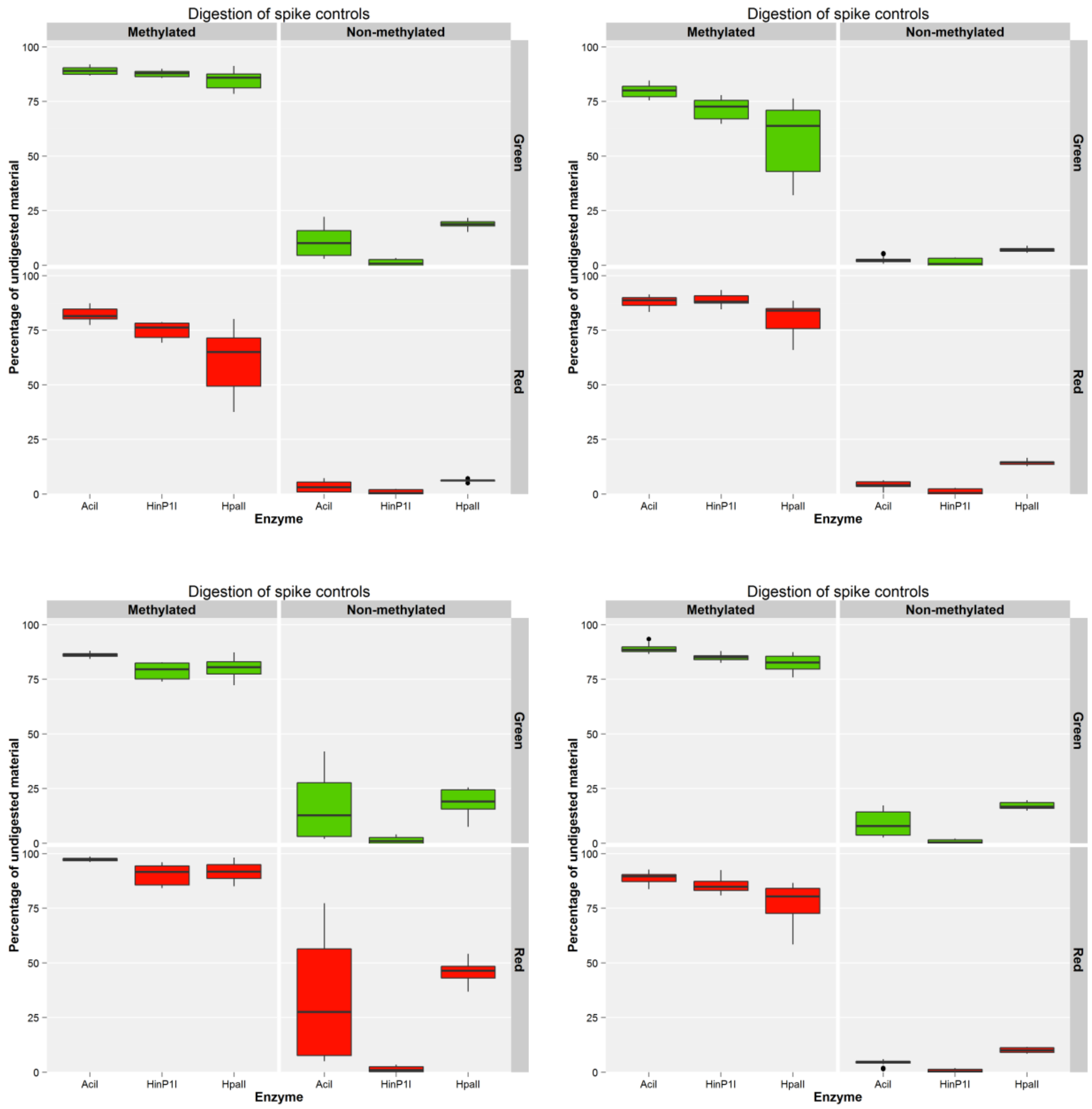

SOV TE spiked-in-control DNA digestion.

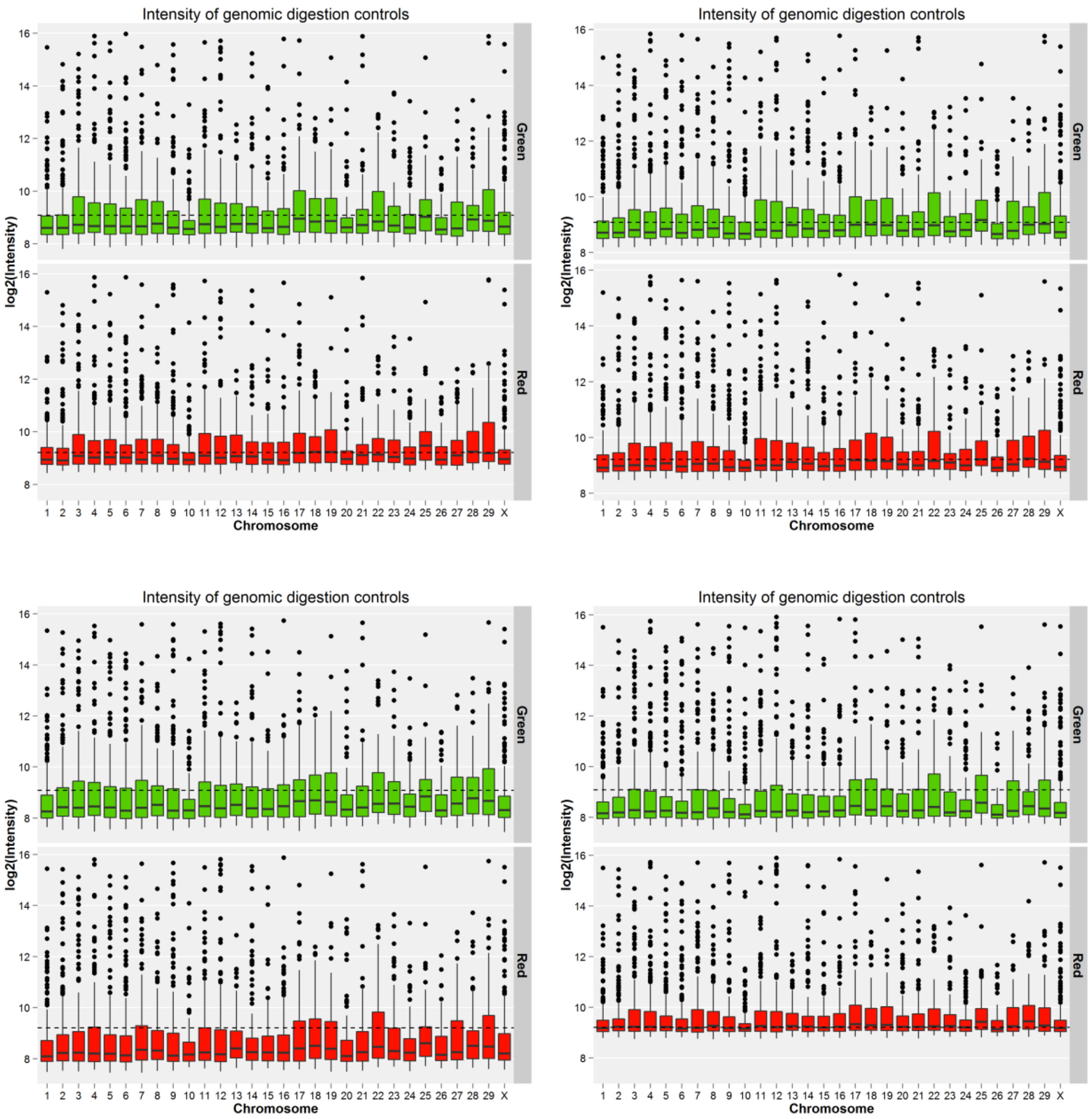

IVP and VIVO genomic DNA digestion controls (TP).

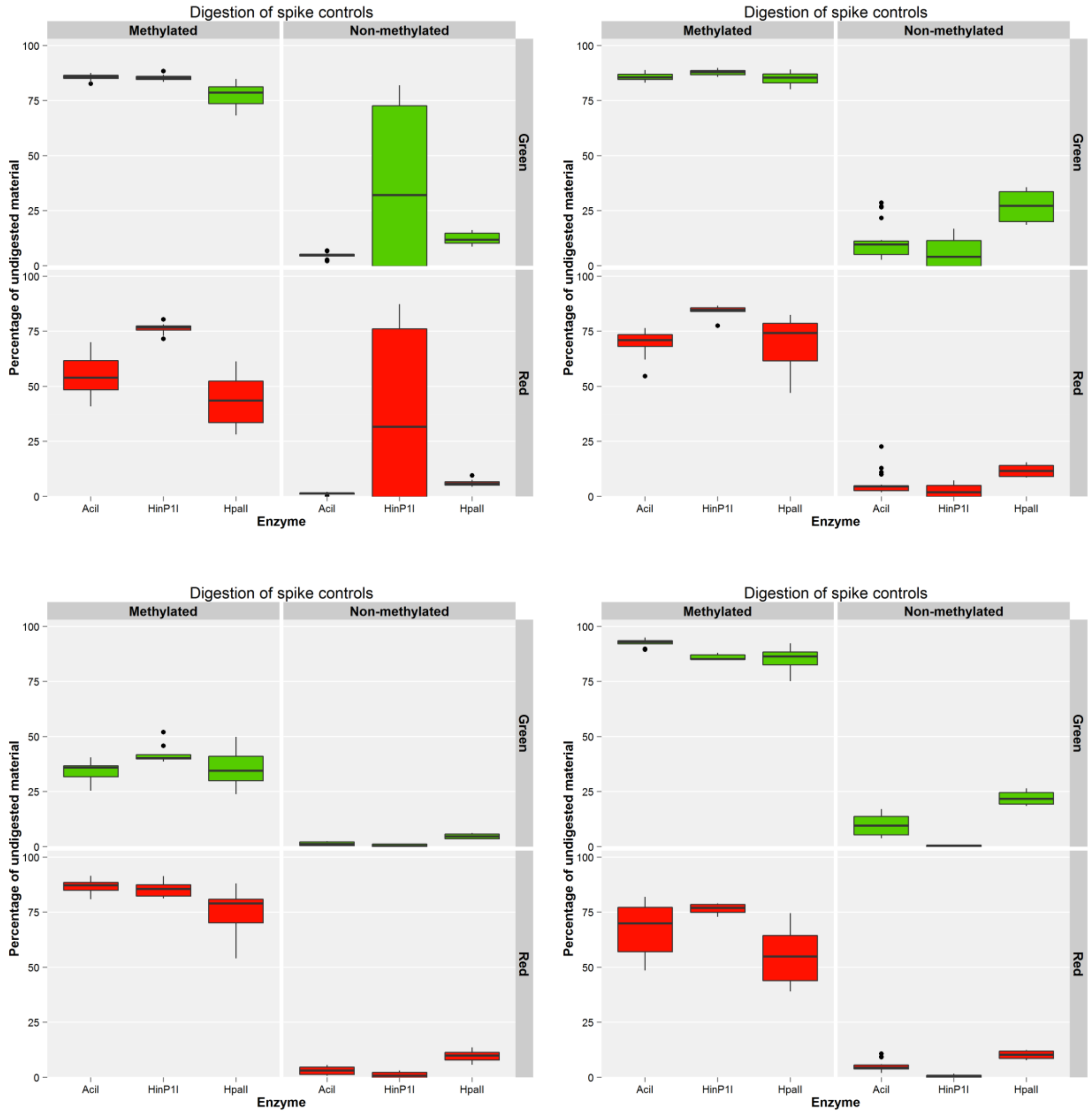

IVP TP spiked-in-control DNA digestion.

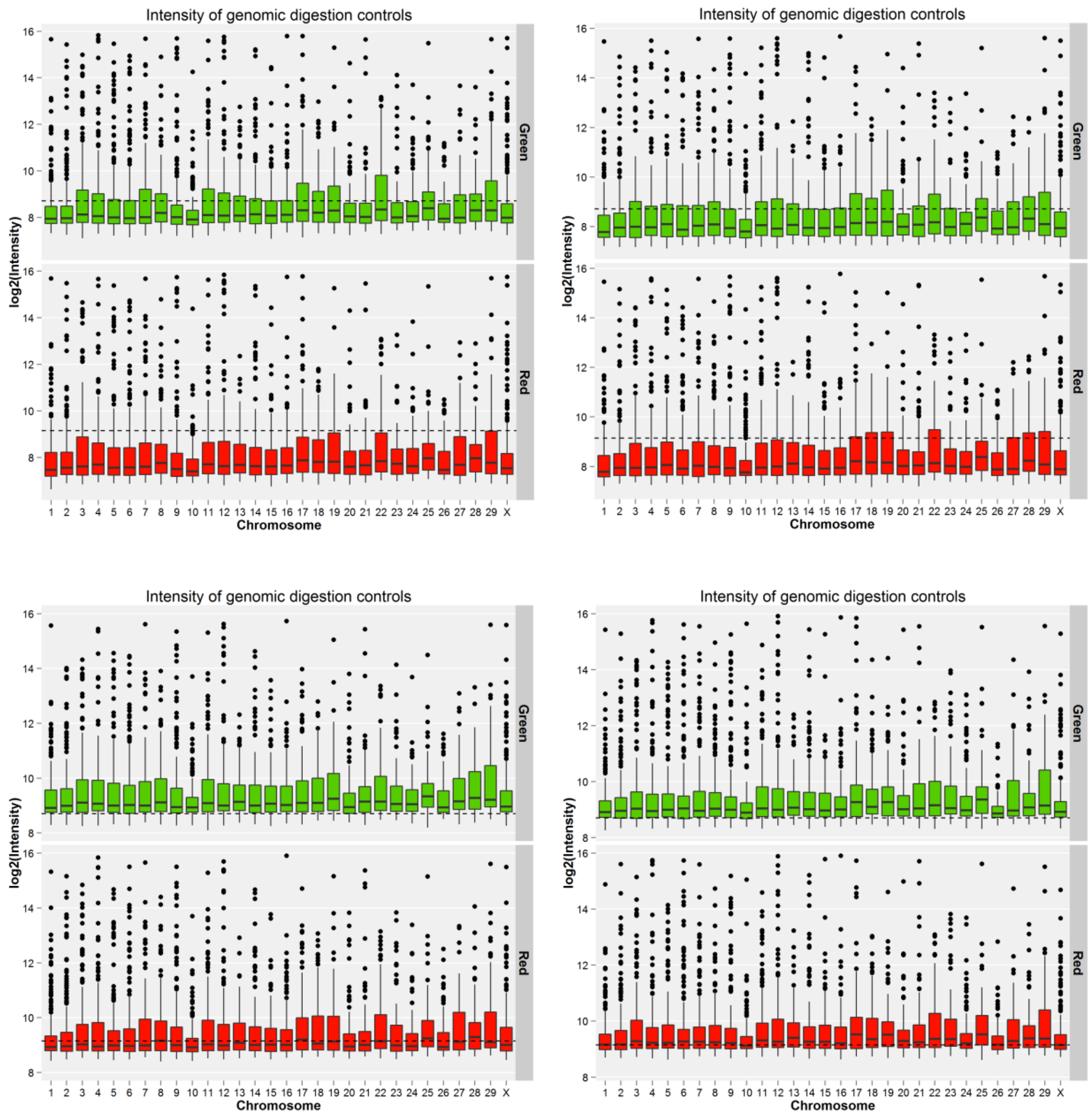

SOV and VIVO genomic DNA digestion controls (TP).

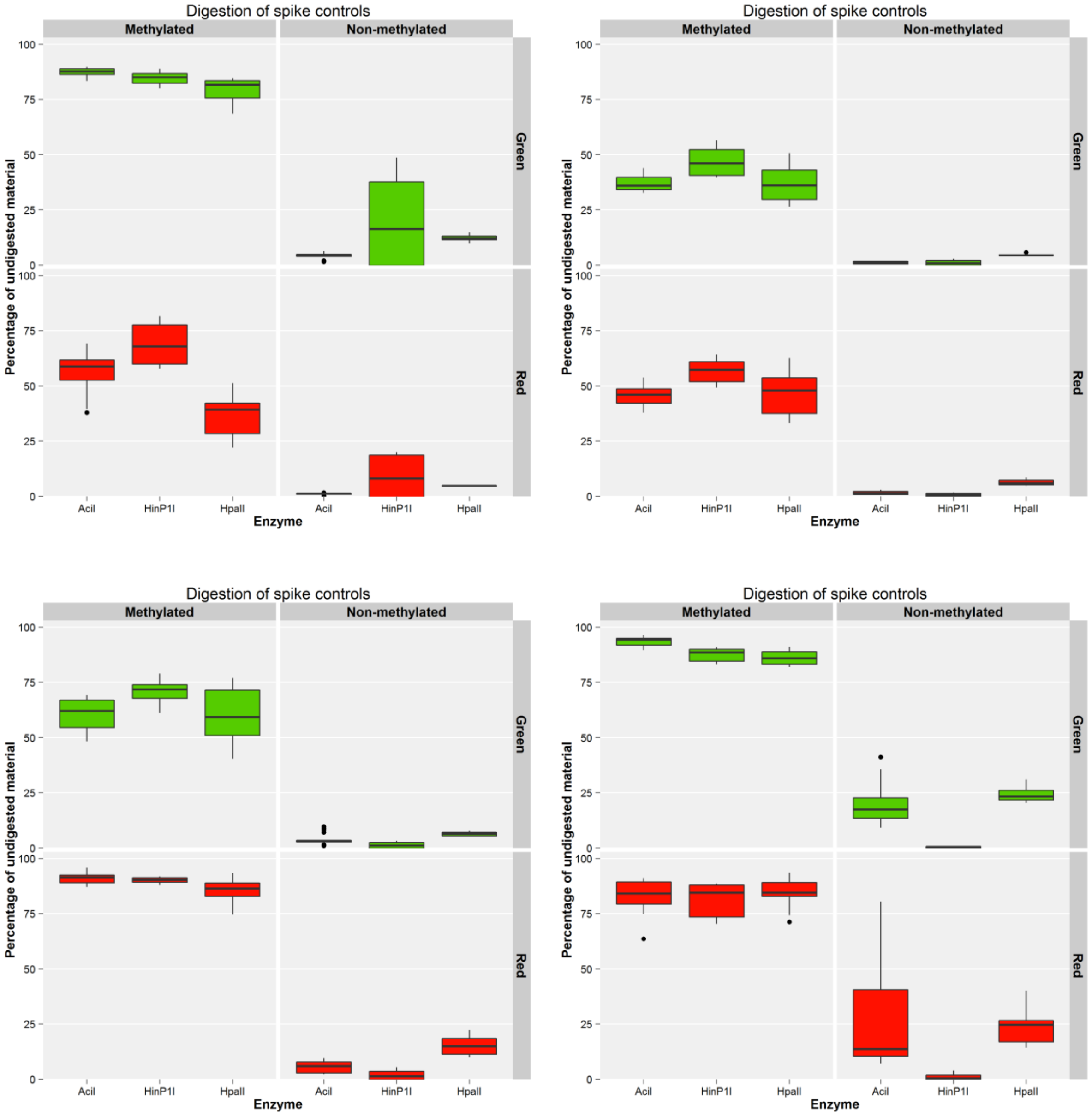

SOV TP spiked-in-control DNA digestion.
