# Supplementary material for: Intragenic sequences in the trophectoderm harbour the greatest proportion of methylation errors in day 17 bovine conceptuses generated using assisted reproductive technologies
Source: BMC Genomics. 2018 Jun 5;19:438. doi: 10.1186/s12864-018-4818-3 (PMC5987443; doi:10.1186/s12864-018-4818-3)
Supplement: Supplementary file 5 — Table S1. Pyrosequencing primers used for array validation. Table S2. Primers used for gene expression analysis. (DOCX 17 kb) [file 12864_2018_4818_MOESM5_ESM.docx]

**Table S1**. Pyrosequencing primers used for array validation.

| Gene ID | Forward Primer 5’ – 3’ | Reverse Primer 5’ – 3’ | Sequencing Primer | NCBI Ref Seq ID |
| --- | --- | --- | --- | --- |
| Pyro |  |  |  |  |
| *RNF7*  (assay 1) | GATTAGTGGTTATTTTGGGGGAAG  ATGA | **[BIO]**CCACACAAAAATCAAAATC  AAAACCCTAAA | ATGTGTGATTAGAATT  ATTAGAT | NM_001075720.2 |
| *RNF7*  (assay 2) | GGGTTTTGATTTTGATTTTTGTGTG | **[BIO]**AACAAAACCATTAAACAAA  ATCATTTCCA | ATTTTTGTGTGGTTTGT | NM_001075720.2 |
| *GLTP* | **[BIO]**ATTAGGTTTTTAGTATTTTGA  TATAGGTAT | CCACCCTCTCATTTCATCTCA | ACTCAAAAACAAAAAC  ATTCT | NM_175799.3 |
| *TRAPPC9* | GAAGGAGGTTGATAGTTTTTATATA  AGG | **[BIO]**CACTCTTCATTTATTATTTT  AATTACCTAT | GGTTGATAGTTTTTATA  TAAGGT | NM_001037474.2 |
| *CRISPLD2* | **[BIO]**ATGAATATAGGGTTTTAAAGG  AGAAGT | CCAAAACTTTCTATTTTAAAAAT  CCCAAT | CTTTCTATTTTAAAAAT  CCCAATAA | NM_001100299.1 |

**[BIO] =** denotes the location of the 5’ biotin label

**Table S2.** Primers used for gene expression analysis.

| Gene ID | Forward Primer 5’ – 3’ | | Reverse Primer 5’ – 3’ | | Amplicon (bp) | NCBI Ref Seq ID | |
| --- | --- | --- | --- | --- | --- | --- | --- |
| qPCR | |  | |  |  | |  |
| *TCEB3* | | CAAGTTGTGGAGAAGCTGCA | | AGGGCGGAGAGTTTCTTCAA | 86 | | NM_001102333.1 |
| *OCRL* | | GATGAAGGGTCCTCTCCGG | | ACACGCTGTTCCTTCTTGTG | 99 | | NM_001102191.2 |
| *ATP1A1* | | GAGGGATATGGACGAGCTGA | | TCGGCTCAAGTCTGTTCCAT | 97 | | NM_001076798.1 |
| *SNRPN* | | CACCTACACCTGTTGGTCGAG | | GTGTCCCACGAGTAGGAGGA | 111 | | NM_001079797.1 |
| *H19* | | GCTGCATTTTGGAACCACTAC | | CTGCTCTTCCGGTGGTGT | 94 | | NR_003958.2 |
